# Economic Evaluation in Global Perspective: A Bibliometric Analysis of the Recent Literature

**DOI:** 10.1002/hec.3305

**Published:** 2016-01-25

**Authors:** Catherine Pitt, Catherine Goodman, Kara Hanson

**Affiliations:** ^1^ Department of Global Health and DevelopmentLondon School of Hygiene & Tropical MedicineLondonUK

**Keywords:** bibliometrics, economic evaluation, cost‐effectiveness analysis, low‐income countries, middle‐income countries, high‐income countries

## Abstract

We present a bibliometric analysis of recently published full economic evaluations of health interventions and reflect critically on the implications of our findings for this growing field. We created a database drawing on 14 health, economic, and/or general literature databases for articles published between 1 January 2012 and 3 May 2014 and identified 2844 economic evaluations meeting our criteria. We present findings regarding the sensitivity, specificity, and added value of searches in the different databases. We examine the distribution of publications between countries, regions, and health areas studied and compare the relative volume of research with disease burden. We analyse authors' country and institutional affiliations, journals and journal type, language, and type of economic evaluation conducted. More than 1200 economic evaluations were published annually, of which 4% addressed low‐income countries, 4% lower‐middle‐income countries, 14% upper‐middle‐income countries, and 83% high‐income countries. Across country income levels, 53, 54, 86, and 100% of articles, respectively, included an author based in a country within the income level studied. Biomedical journals published 74% of economic evaluations. The volume of research across health areas correlates more closely with disease burden in high‐income than in low‐income and middle‐income countries. Our findings provide an empirical basis for further study on methods, research prioritization, and capacity development in health economic evaluation.

## 1 Introduction

In 2012, Wagstaff and Culyer published a high‐profile bibliometric analysis that set out to characterise the entirety of the health economics field, updating and extending prior work by Rubin and Chang (2003). Their ambitious work examined publications across 42 years (1969–2010) and generated much discussed rankings of the leading authors, institutions, and topics of health economics research over time. By restricting their analyses to journals indexed in EconLit, however, they omitted the substantial body of health economics research published in the medical literature, including many economic evaluations of health interventions. This important and growing area of health economics examines the relative efficiency of alternative courses of action in improving health (Drummond *et al*., 2005).

To address this gap, we present a bibliometric analysis of recently published, full health economic evaluations (Drummond *et al*., 2005) and reflect critically on the implications of our findings. Bibliometric analysis is defined as the quantitative study of written communication in forms such as journal articles and books (Pritchard, 1969). It sets out to characterise a literature, rather than examine the findings of that literature, which is the approach of a systematic review. We stratify our analyses of the economic evaluation literature by the income group classification of the countries studied (World Bank, 2015). This stratification ensures that findings regarding low‐income and middle‐income countries (LMICs) receive due attention, given that they are home to 84% of the world's population and bear 89% of the global burden of disease (GBD) (World Health Organization (WHO), 2014). In light of the growing interest in global health and priority setting, this contribution to the evidence base is also timely.

A previous bibliometric analysis of cost‐effectiveness analyses (CEAs) was limited to studies reporting outcomes as cost per quality‐adjusted life‐year (QALY) up to 2006 published in English in journals indexed in Medline (Greenberg *et al*., 2010). As QALYs were infrequently used in LMICs up to 2006, this restriction biased Greenberg *et al*.'s findings towards studies undertaken in HICs and omitted nearly half of full economic evaluations (as we will show). Much has also changed since 2006, with a rapid expansion in the literature, including in LMICs.

By 1984, just a handful of economic evaluations of health interventions had been conducted in LMICs (Mills and Thomas, 1984) and even in 2000, Walker and Fox‐Rushby (2000) were still able to review critically the 107 economic evaluations of interventions to address communicable diseases in LMICs published between 1984 and 1997. In the past decade, however, the body of work has expanded such that it has been possible for reviews to focus on specific disease areas, for example non‐communicable diseases (Mulligan *et al*., 2006); road traffic injuries (Waters *et al*., 2004); malaria (Goodman and Mills, 1999, White *et al*., 2011); various aspects of HIV/AIDS (Creese *et al*., 2002, Galarraga *et al*., 2009, Walensky *et al*., 2010, Johri and Ako‐Arrey, 2011) and tuberculosis (Fitzpatrick and Floyd, 2012, Chavan *et al*., 2011); vaccination for Haemophilus influenzae type b (Griffiths and Miners, 2009), seasonal (Ott *et al*., 2013) and pandemic influenza (Perez Velasco *et al*., 2012); human papilloma virus (Natunen *et al*., 2013, Fesenfeld *et al*., 2013); cardiovascular diseases (Suhrcke *et al*., 2012); surgery (Chao *et al*., 2014); and strategies to improve the demand and supply of maternal and neonatal care (Mangham‐Jefferies *et al*., 2014). Reviews of economic evaluations in LMICs have also narrowed their focus by geography, for example to Meso‐America (Valencia‐Mendoza *et al*., 2011), Latin America and the Caribbean (Augustovski *et al*., 2009), Thailand (Teerawattananon *et al*., 2007), Nigeria (Gavaza *et al*., 2010), Tanzania (Mori and Robberstad, 2012), and Ghana (Odame, 2013). In adopting a more constrained perspective, these reviews have allowed important insights into the economic evidence for specific disease areas or geographies, but have not provided a wider perspective on the overall economic evaluation literature in LMICs, nor been able to compare this literature with the far larger body of economic evaluations in high‐income countries (HICs).

We aim to provide a recent snapshot of the state of the economic evaluation field. In the following sections, we describe the methods for generating and analysing our data, present our results, and reflect on the state of the field and the implications of our findings for research priority setting and capacity development.

## 2 Methods

We began by developing a comprehensive database of peer‐reviewed research articles reporting a primary, full economic evaluation. Following Drummond *et al*. (2008), we defined ‘full economic evaluation’ as studies which evaluate the efficiency of alternative interventions or courses of action by combining data on the costs and effects on human health of the alternatives in CEA, cost‐utility analysis (CUA), or cost‐benefit analysis (CBA). Further, we aimed to restrict our database to articles which went beyond simple reporting of some cost and effect data, and instead included only articles which either (i) produced a summary measure of efficiency, such as a ratio (e.g. incremental cost‐effectiveness ratio), probability (e.g. that an intervention is cost‐effective given a defined threshold), difference (e.g. incremental net benefit), and/or graph, such as a cost‐effectiveness plane or cost‐effectiveness acceptability curve as recommended in International Society for Pharmacoeconomics and Outcomes Research guidelines (Ramsey *et al*., 2005), or (ii) which demonstrated strict dominance (i.e. that one intervention is both more costly and less effective than the other). We defined ‘primary research’ to include the production of a novel estimate (i.e. to include modelling studies) and to exclude reviews which only cite previously published estimates.

Our analysis was restricted to articles published from 1 January 2012 to the date of our searches, 3 May 2014, comprising a period of 28 months. This restriction reflects both our aim to provide a recent snapshot of a rapidly changing field and also practical considerations, since even this restricted timeframe required screening, cleaning, and coding large volumes of data. In the following sections we describe the process of constructing the database and our analytical methods.

### 2.1 Data

#### Search strategies.

Figure S1 illustrates our search strategy in a flow diagram adapted from the PRISMA guidelines for systematic reviews.(Liberati *et al*., 2009) We identified 17 potential databases for our search by consulting recent systematic reviews of economic evaluations and a health sciences librarian to identify databases which seemed, *prima facie*, to be potentially useful or used by researchers.

Based on preliminary searches in all databases and a review of their content and functionality, we selected 14 databases for our final search: two health economics databases (the National Health Service Economic Evaluations Database (NHS EED) and the Health Economic Evaluations Database (HEED)), one economics database (EconLit), one general literature database (Scopus), two broad databases (the Science Citation Index Extended (SCI), and the Social Science Citation Index, which were searched simultaneously), and eight health sciences databases (Embase, Medline including in‐process, Latin American Health Sciences Literature (LILACS), Global Health, PsycInfo, Scielo, Biosis, and Cinahl). We excluded Google Scholar because Google prohibits bulk downloading of citations; Pubmed because we were able to obtain the same set of articles (Medline, Medline‐in‐process, and Pubmed‐not‐Medline) in our search using the Ovid SP interface, which we also used to access EconLit, Embase, Global Health, and PsycInfo, and the Tufts Cost‐Effectiveness Analysis Registry because its coverage was limited to articles published in English which report outcomes as QALYs and it charges substantial access fees.

Search strategies were optimised individually for each database, taking into account the scope of each database and the features of its user interface. Careful checks were performed to ensure that the initial search was as sensitive as possible and that any restrictions increased specificity without compromising sensitivity. Each time we considered an additional restriction to increase the specificity of the search, such as excluding all articles with the word ‘protocol’ in the title, we first reviewed the first 100 excluded records, and revised the search strategy if any excluded records were found to meet our inclusion criteria. Full details of the final search strategy employed in each database are provided in Table S1 and further discussion of the reasons for not using controlled vocabulary indexing terms (e.g. MeSH terms) is available in Text S1.

#### Merging and screening.

Search results were exported to Excel. We identified duplicate records to produce a set of unique records linked to the bibliographic data in all of the databases in which they were found. By comparing multiple databases and carefully reviewing data, we corrected many of the errors within the bibliographic data. Titles and, if necessary, abstracts and in some cases full text were screened by one author (CP) to determine whether they met our inclusion criteria. Although only English‐language search terms were used, no language restrictions were applied. Keyword searches of all text fields were used to facilitate identification of articles for exclusion (using terms such as ‘review’ and ‘protocol’) and inclusion (using terms such as ‘dominant’ and ‘cost‐utility’).

We excluded articles which described themselves as CEA, CUA, or CBA but did not meet our inclusion criteria. For example, self‐proclaimed ‘cost‐benefit analyses’ which only compared the costs of interventions with cost savings resulting from reduced subsequent health care use were excluded as they did not measure health benefits. Cost‐minimization analyses were similarly excluded (Dakin and Wordsworth, 2013), as were the many articles declaring an intervention ‘cost‐effective’ which did not analyse both costs and effects.

### 2.2 Analyses

All analyses are disaggregated by country income group and were conducted in Microsoft Excel.

#### Databases.

For each of the 14 databases, we provide estimates of the sensitivity 1Sensitivity = (number of economic evaluations identified by our search of the given database) / (total number of economic evaluations identified in our final economic evaluation database). and specificity 2Specificity = (number of economic evaluations identified by our search of the given database) / (total number of records identified by our search of the given database). of our search. Given the substantial overlap between databases and to allow us to identify the minimum number of databases required to achieve a given overall sensitivity, we also assessed the added value of each database firstly, by identifying the database yielding the greatest number of economic evaluations, and secondly, by ranking the remaining databases in descending order according to the number of *additional* economic evaluations they identified beyond those already identified by a more highly ranked database.

#### Geographical areas studied.

Key term searches were developed to classify articles by country (or countries) studied, which were then mapped onto World Bank income groups and regions (World Bank, 2015). 3Macao, Hong Kong, and Taiwan, which are all classified as high‐income countries by the World Bank, were analysed separately from the mainland of the People's Republic of China, an upper‐middle‐income country. All potentially ambiguous country names were reviewed, 4Potentially ambiguous country names included for example, ‘Congo’, ‘Korea’, ‘Niger’, and ‘Guinea’, each of which is contained within more than one country name; ‘China’, which is often used in reference to Taiwan, Hong Kong, and Macao; ‘Japan’, which appears within the bibliographic data of studies of Japanese encephalitis; and ‘England’, which may refer to the United Kingdom, to New England in the USA, or to studies published in the New England Journal of Medicine. as were all articles not classified by any search term or classified as analysing multiple income groups. Articles which described themselves as studying a region or set of countries (such as ‘malaria endemic countries’ (WHO Global Malaria Programme, 2014)) were classified according to all the countries within that region. A single article could be classified as belonging to multiple income levels or regions.

#### Health areas.

We developed a classification of 25 health areas so as to allow comparability with the global burden of disease (GBD) estimates (WHO, 2014), to be implementable with an electronic key term search, and to permit meaningful analysis. In Table S2, we show how our 25 health areas map onto the GBD and onto the WHO's International Classification of Disease, version 10 (WHO, 2011). A set of up to 49 search terms was developed for each of our health areas through an iterative process.

As with countries studied, a single article could be classified as belonging to multiple health areas. For example, we counted economic evaluations of interventions for gestational diabetes as both ‘maternal and newborn health’ and ‘diabetes’, and interventions to address HIV and tuberculosis co‐infection (Pawlowski *et al*., 2012) as addressing each disease. While this could be considered double‐counting, we argue that interventions addressing multiple areas do not contribute any less to each area than those interventions addressing only one disease. Further information is available in Text S2.

We then compared the distribution of health areas studied in economic evaluations to the GBD. Comparisons are presented graphically with scatter plots comparing the volume of economic evaluations and burden of disease by (i) ranking and (ii) proportion of total, disaggregated by income group and in total, which allows us both to assess the correlation and to identify health areas which are outliers meriting deeper exploration.

#### Languages and journals.

Journals were classified as follows: (i) biomedical; (ii) health economics, services, policy, and/or social sciences; or (iii) other (Table S3). We analysed the proportion of health economic evaluations published in each journal type, the top 20 journals, and the concentration of economic evaluations by income group and in total.

The language of the full text was also analysed. Where the full text was available in English and another language, the article was categorised as English to permit analysis of what would be missed if only English‐language publications were considered. As there were many errors in the language data in the bibliographic databases, these data were also compared with the journal name and country studied, and in some cases the full text or journal website examined, to arrive at a final language classification.

#### Types of economic evaluation.

We used key term searches to disaggregate studies by self‐reported type: CBA, CUA, and other CEAs. We further disaggregated cost‐utility studies between those employing disability‐adjusted life‐years (DALYs) and those employing QALYs. Search terms are listed in Table S4.

#### Institutional and geographical affiliations of authors.

We analysed data on the institutional affiliation of all authors to develop a comprehensive picture of the institutions and countries contributing to health economic evaluations.

We identified the top 10 institutions within each income group by volume of economic evaluations produced. As in previous work (Wagstaff and Culyer, 2012, Rubin and Chang, 2003), schools, colleges, and institutes were aggregated with the university to which they belonged, with the exception of the highly federal Universities of London, California, Texas, and other similar university systems, whose constituent members were analysed separately.

We considered a number of possible approaches for analysing articles with more than one institutional affiliation, including assigning a fractional value (and even weighted fractional values reflecting author order) to each institution based on the number of authors or institutions represented on a given article (Aksnes *et al*., 2012, Hagen, 2013, Retzer and Jurasinski, 2009). However, we rejected such approaches because using zero‐sum metrics, in general, establishes a perverse incentive against collaboration between institutions and against the crediting of collaborators. We therefore assigned one point per institution per article, regardless of the number of institutions or authors on a given article. This has the disadvantage of weighting the analysis towards articles from multiple institutions, as these articles are counted multiple times in the analyses of institutional and country affiliations. More information on how we classified health areas and institutional affiliations is available in Text S2.

## 3 Results

### 3.1 Search results

In total, our searches of the 14 databases identified 47 407 records (Figure S1). After duplicate removal, 15 057 unique records remained, and after screening, a total of 2844 unique, full economic evaluations were retained for analysis.

### 3.2 Databases

Our search of Scopus identified the largest number of economic evaluations (*n* = 2409), 85% of our total, followed by NHS EED, which identified 80% of the articles we identified (Table S5). Together, these two databases identified 96% of articles, and adding the Medline search increased this to 98%. With each additional database, the incremental gains were diminishingly small, and one database, Lilacs, failed to identify any additional articles beyond those identified by other databases. Econlit identified just 42 economic evaluations, 1% of the total. If we exclude NHS EED from consideration as it ceased to update records from March 2015 and exclude Wiley HEED as it ceased to be available from the end of 2014, our searches of a combination of Scopus, Medline, and Global Health would identify 91% of the economic evaluations, but a remaining 7% of economic evaluations in our database were only identified by NHS EED and Wiley HEED and not by our searches of other databases (Table S6). If we restrict the analysis to articles studying LMICs and exclude NHS EED and Wiley HEED, our searches of Scopus, Medline, and Global Health would together identify 93% of economic evaluations in LMIC settings, while 4% were only identified in NHS EED and Wiley HEED (Table S7).

### 3.3 Subjects studied

#### Geographical areas studied.

At least one country, region, and income group studied was assigned to all economic evaluations identified. Of these, 83% studied HICs, 14% upper‐MICs, 4% lower‐MICs and 4% LICs. These sum to more than 100% because 2% of articles reported studies set in multiple countries in more than one of the four income groups. As expected, most articles reported findings from Europe and Central Asia (44%) and/or North America (34%) (Table 1).

**Table 1:** Number of economic evaluations by income group and region of study

	Income group(s) of countries studied						
Region(s) studied	Low	Lower‐middle	Upper‐middle	High	Multiple^a^	Total	% of total
East Asia and Pacific	22	43	165	229	25	405	14%
Europe and Central Asia	11	16	44	1210	20	1243	44%
Latin America and Caribbean	13	18	116	16	19	129	5%
Middle East and North Africa	14	20	43	27	20	62	2%
North America	1	1	1	960	1	960	34%
South Asia	27	49	20	15	25	56	2%
Sub‐Saharan Africa	92	64	78	22	46	158	6%
Multiple^a^	27	35	31	85	38	102	4%
Total	104	121	391	2350	63	2844	100%
% of total	4%	4%	14%	83%	2%	100%	

a Articles studying at least two countries of differing income levels or regions are categorised as ‘Multiple’.

**Figure 1:**
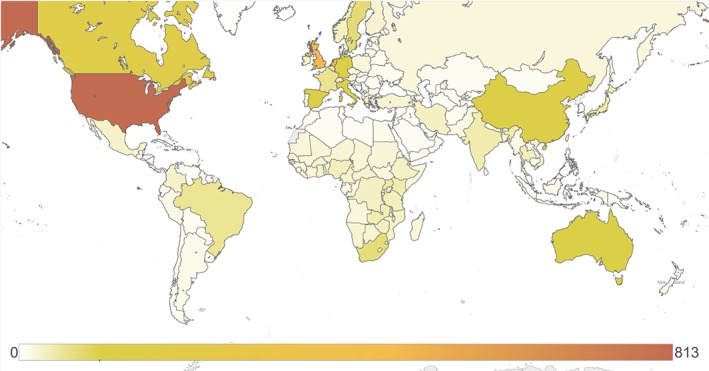
Number of economic evaluations set in each country. The intensity of shading reflects the number of economic evaluations analyzing each country over the 28‐month period from 1 January 2012 to 3 May 2014

Table 2 and Figure 1 present the individual countries most frequently studied. The United States (USA) was the subject of 813 studies, followed by the United Kingdom (UK) (*n* = 478) and six further countries which were each studied in at least 100 articles. While China, South Africa, and Brazil were studied in a relatively large number of articles, only 10 upper‐MICs were studied in at least 20 articles each. Led by Uganda, India, Kenya, and Zambia, all of the top 20 LICs and lower‐MICs were studied in more than 20 economic evaluations, in part because 61 of the 184 articles (33%) studying at least one LIC or lower MIC examined more than one country and 33 LIC and lower MIC articles (18%) studied more than 10 countries. In upper‐MICs and HICs, only 14% (*n* = 54) and 7% (*n* = 169) of studies, respectively, examined more than one country and 8% (*n* = 32) and 1% (*n* = 27) examined more than 10 countries.

**Table 2:** Top 20 countries most frequently studied in economic evaluations by income group

	High income			Upper‐middle‐income			Low and lower‐middle‐income		
Rank	Country	N	%	Country	N	%	Country	N	%
1	USA	813	35%	China	116	30%	Uganda	49	27%
2	UK	478	20%	South Africa	71	18%	India^a^	41	22%
3	Netherlands	183	8%	Brazil	56	14%	Kenya^a^	41	22%
4	Canada	162	7%	Thailand	36	9%	Zambia	39	21%
5	Spain	136	6%	Iran	31	8%	Malawi	35	19%
6	Germany	109	5%	Colombia^a^	28	7%	Nigeria^a^	34	18%
7	Australia	100	4%	Mexico^a^	28	7%	Tanzania^a^	34	18%
8	Italy	98	4%	Turkey	24	6%	Zimbabwe	33	18%
9	Sweden	74	3%	Botswana^a^	23	6%	Congo, Dem. Rep.	30	16%
10	France	57	2%	Namibia^a^	23	6%	Ethiopia	29	16%
11	Japan	45	2%	Angola	18	5%	Lesotho^a^	28	15%
12	Belgium	42	2%	Gabon	17	4%	Mozambique^a^	28	15%
13	Denmark	33	2%	Mauritius^a^	14	4%	Rwanda^a^	28	15%
14	Korea, Rep.^a^	31	1%	Peru^a^	14	4%	Vietnam^a^	28	15%
15	Norway^a^	31	1%	Seychelles^a^	14	4%	Ghana	27	15%
16	Greece	29	1%	Bulgaria	13	3%	Central African Republic	26	14%
17	Ireland	27	1%	Argentina^a^	12	3%	Burundi^a^	25	14%
18	Switzerland^a^	24	1%	Hungary^a^	12	3%	Cameroon^a^	25	14%
19	Finland^a^	24	1%	Maldives	11	3%	Eritrea^a^	25	14%
20	Taiwan	23	1%	Serbia	10	3%	Burkina Faso	24	13%
High‐income countries		2350	100%	Upper‐middle‐income countries	391	100%	Low‐ and lower‐middle‐income countries	184	100%

a Equal ranking with country above and/or below.

#### Health areas studied and the global burden of disease.

At least one health area was assigned to 2829 (99.5%) articles. The mean number of health areas per article was 1.4 and the maximum 7. Whereas 71% of articles were assigned a single health area, 21% addressed two health areas and 8% addressed three or more. In LICs, three health areas dominate: HIV/AIDS (30% of classified LIC articles), neonatal and maternal conditions (16%), and malaria (15%) (Table 3). In lower‐MICs, HIV/AIDS again dominates (23%), but the remaining health areas are more evenly distributed; malaria comes second (11%), and is followed by other infectious diseases (8%) and mental health (8%); half of the latter focused on HIV treatment and prevention amongst injection drug users. In upper‐MICs, HIV/AIDS (12%) falls to second place, while cancer and other neoplasms (19%) occupy the top spot with cardiovascular (11%) and respiratory diseases (10%) in third and fourth respectively. As HICs are studied in 83% of economic evaluations, the disease areas addressed in economic evaluations in HICs drive the distribution of all economic evaluations conducted worldwide, with cardiovascular diseases (19% in HICs), cancer and other neoplasms (18%), mental health (10%), and musculoskeletal diseases (10%), the leading areas of study in HICs and overall (Table 3).

**Table 3:** Number of economic evaluations by health area and income group

	Income group studied				
Health area	Low	Lower‐middle	Upper‐middle	High	World
Cancer and other neoplasms	7	8	73	416	492
Cardiovascular diseases	3	7	44	448	490
Mental health, cognition, and developmental and behavioural disorders (including self‐harm and substance disorders)	1	10	21	243	268
Musculoskeletal diseases (including back pain)	2	3	18	240	262
Respiratory diseases	6	8	39	188	228
Genitourinary diseases, contraception & fertility	4	4	18	180	203
Other infectious diseases (including encephalitis, hepatitis, other parasitic and vector‐borne diseases, and nematode infections)	6	10	38	111	159
Digestive disorders	3	3	21	127	152
Neonatal and maternal conditions	17	7	23	102	142
HIV/AIDS	31	27	46	61	136
Diabetes	1	3	22	102	125
Malnutrition (including obesity and exercise)	6	4	9	98	113
Wounds and injuries (including violence)	4	7	13	91	109
Endocrine, blood, and immune disorders (excluding diabetes or HIV)	0	1	12	86	99
Neurological conditions	1	3	16	81	98
Skin and oral conditions	0	3	5	67	75
Sense organ diseases	2	3	11	56	68
Tuberculosis	8	9	28	34	62
Sexually transmitted diseases (excluding HIV)	2	1	10	39	49
Diarrhoeal diseases	6	7	9	29	46
Communicable childhood diseases	2	5	9	24	40
Malaria	16	13	8	1	24
Congenital anomalies	0	1	2	20	23
Anaemia	0	1	1	9	11
Meningitis	2	2	3	3	9
TOTAL	104	120	390	2337	2829

A single economic evaluation may address more than one health area in countries of more than one income group. The totals exclude the 15 articles (0.5%) in our data set which could not be classified by health area.

**Figure 2:**
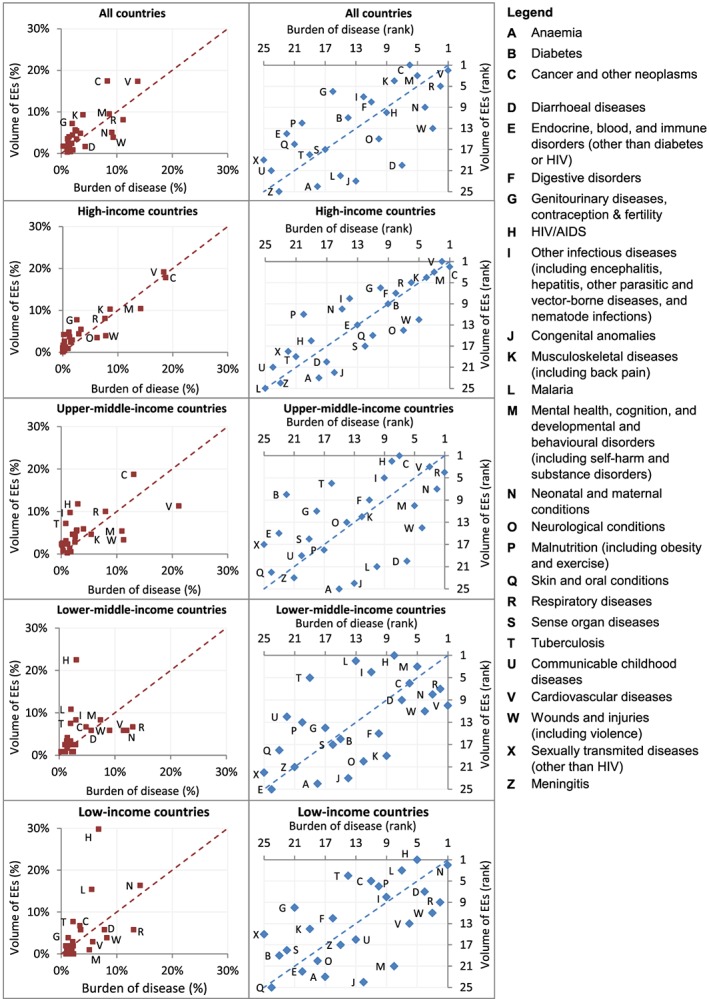
Economic evaluations versus burden of disease by income group. Results are presented in two ways: the lefthand column compares the proportion of the total number of economic evaluations examining each of the 25 health area with the proportion of the total burden of disease accounted for by each health area and the righthand column compares the ranking of the health areas by the volume of economic evaluations and by burden of disease

The distribution of articles across health areas corresponds substantially but by no means perfectly with the global disease burden. The degree of correlation varies by income level, but also depends on whether rankings or proportions are compared. By either metric, the health areas studied in HICs correlate surprisingly well with disease burden and substantially better than economic evaluations in other income groups, which feature more numerous and extreme outliers (Figure 2). The correlation between the health focus of economic evaluations and disease burden is also substantially stronger in studies of HICs than globally, because most economic evaluations (83%) address HICs and are well correlated with HICs' disease burden, whereas most of the GBD (89%) affects LMICs.

HIV/AIDS is studied in a greater proportion of economic evaluations at every income level than its share of the disease burden; however, the gap is much smaller in HICs than in LICs and lower‐MICs, where it is an extreme outlier. Other such ‘winners’ across all income levels include ‘other infectious diseases’; ‘genitourinary diseases, contraception, and fertility’; and ‘sexually transmitted diseases (excluding HIV)’. By contrast, interventions to address wounds and injuries and, to a somewhat lesser extent, neurological conditions, appear to be substantially under‐researched relative to disease burden at every income level.

### 3.4 Journals and languages

Economic evaluations were published in a total of 967 different journals (Table S8). Five hundred fifty‐nine journals published only one economic evaluation each in the entire 28‐month period we analysed and 165 journals published only two. Whereas 802 different journals published HIC articles, only 44 published LIC articles. The proportion of articles published in the top 20 journals for each income group increased steeply down the income groups: 29% of articles studying HICs were published in the top 20 journals publishing HIC evaluations, while 38, 66, and 77% of articles studying upper‐MICs, lower‐MICs, and LICs, respectively, were published in the top 20 journals publishing evaluations set in each of the respective income groups.

**Figure 3:**
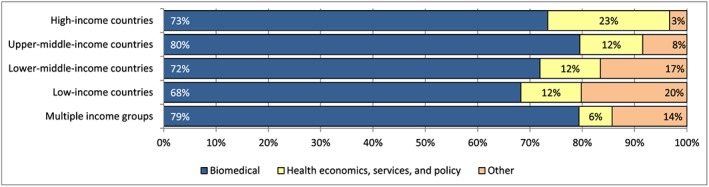
Proportion of economic evaluations by journal type and income group. The classification of journals by type is provided in Web appendix 6. Articles are disaggregated by the income group(s) of the country or countries studied

Overall, 74% of articles were published in biomedical rather than health economics, systems, and policy journals (22%) or other journal types (5%) (Figure 3). In HICs, 6 of the top 10 journals were health economics, systems, or policy journals, compared with only 3 of the top 10 journals publishing articles about LICs and lower‐MICs (Table 4). The top outlet for economic evaluations across all income levels was *PLoS ONE*, an open‐access journal publishing ‘primary research from any scientific discipline’, which ranked amongst the top three journals for all income groups. *Vaccine* ranked fourth overall (*n* = 66) and in the top five for all income groups. Yet overall, journals tended towards segregation by income group; 6 of the top 10 journals publishing economic evaluations about HICs did not publish a single LIC or lower MIC study and two of the remaining published only one each.

**Table 4:** Journals publishing the greatest number of economic evaluations by income group of countries studied

	Low and lower‐middle			Upper‐middle			High			All		
Rank	Journal	Type	N	Journal	Type	N	Journal	Type	N	Journal	Type	N
1	PLoS One	Other	30	PLoS One	Other	31	Journal of Medical Economics	HEPS	100	PLoS One	Other	121
2	Vaccine	BM	13	Vaccine	Other	17	Health Technology Assessment	HEPS	82	Journal of Medical Economics	HEPS	101
3	Malaria Journal	BM	9	Value in Health Regional Issues	BM	11	PLoS One	Other	70	Health Technology Assessment	HEPS	82
4	Journal of Acquired Immune Deficiency Syndromes	BM	8	Value in Health	HEPS	8	Value in Health	HEPS	54	Vaccine	BM	66
5	Health Policy and Planning	HEPS	8	BMJ	HEPS	7	Vaccine	BM	44	Value in Health	HEPS	63
6	BMJ	BM	6	AIDS	BM	7	ClinicoEconomics and Outcomes Research	HEPS	36	ClinicoEconomics and Outcomes Research	HEPS	37
7	Value in Health Regional Issues	HEPS	6	Cadernos de Saúde Pública	BM	7	European Journal of Health Economics	HEPS	35	European Journal of Health Economics	HEPS	36
8	Cost Effectiveness and Resource Allocation	HEPS	5	BMC Public Health	BM	6	PharmacoEconomics	HEPS	33	PharmacoEconomics	HEPS	34
9	PLoS Medicine	BM	5	BMC Health Services Research	BM	6	Clinical Therapeutics	BM	28	Clinical Therapeutics	BM	32
10	AIDS	BM	4	PLoS Medicine	HEPS	5	BMJ Open	BM	26	Value in Health Regional Issues	HEPS	28
11	PloS Neglected Tropical Diseases	BM	4	International Journal of Tuberculosis and Lung Disease	BM	5	Applied Health Economics and Health Policy	HEPS	26	BMJ Open	BM	26
12	BMC Public Health	BM	3	Journal of the Medical Association of Thailand	BM	5	International Journal of Technology Assessment in Health Care	HEPS	22	Applied Health Economics and Health Policy	HEPS	26
13	International Journal of Tuberculosis and Lung Disease	BM	3	Malaria Journal	BM	4	Cancer	BM	21	International Journal of Technology Assessment in Health Care	HEPS	25
14	World Journal of Surgery	BM	3	Journal of Acquired Immune Deficiency Syndromes	BM	4	BMJ	BM	19	BMC Health Services Research	HEPS	23
15	Bulletin of the World Health Organization	HEPS	3	Cost Effectiveness and Resource Allocation	BM	4	BMC Health Services Research	HEPS	17	Cancer	BM	21
16	Tropical Medicine and International Health	BM	3	Clinical Therapeutics	HEPS	4	American Journal of Managed Care	BM	16	BMJ	BM	20
17	Clinical Infectious Diseases	BM	2	BMC Infectious Diseases	BM	4	Osteoporosis International	BM	14	BMC Public Health	BM	20
18	Lancet	BM	2	Revista Panamericana de Salud Pública	BM	4	Gynecologic Oncology	BM	14	Cost Effectiveness and Resource Allocation	HEPS	20
19	Biosystems	BM	2	Modern Preventive Medicine	BM	4	BMC Public Health	BM	13	American Journal of Managed Care	BM	16
20	Journal of Pediatrics	BM	2	Biomedica	BM	4	Cost Effectiveness and Resource Allocation	HEPS	13	AIDS	BM	16
	Lancet Global Health	BM	2	Chinese Journal of New Drugs	BM	4	BJU International	BM	13			
	Proceedings of the National Academy of Sciences of the USA	BM	2	Zhonghua liu xing bing xue za zhi	BM	4	Heart	BM	13			
	Journal of the Pakistan Medical Association	BM	2									
	Disasters	Other	2									

BM: Biomedical; HEPS: Health economics, policy, and services; OTH: Other.

All articles addressing LICs and lower‐MICs were published in English, while 4% of HIC articles (*n* = 89) were published in other languages, as was a striking 22% (*n* = 87) of all articles addressing upper‐MICs. In upper‐MICs, Chinese was the leading non‐English language (*n* = 48, 12%), followed by Spanish (23, 6%), Portuguese (*n* = 13, 3%), Turkish (*n* = 2, 1%), and Farsi (*n* = 1, 0%), while in HICs, Spanish was the language of full‐text for 46 articles (2%), followed by German (*n* = 13, 1%), and 10 other languages.

### 3.5 Types of economic evaluation

**Figure 4:**
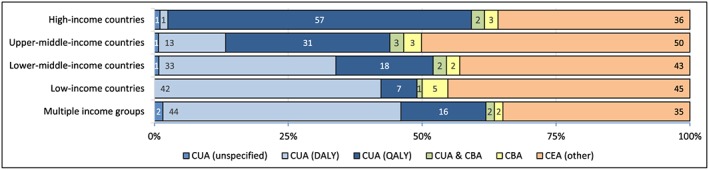
Proportion of economic evaluations by analytical type and income group studied. In this figure, ‘cost‐effectiveness analysis’ refers to articles meeting our definition of a full economic evaluation but not containing any keywords to define it more specifically as a cost‐utility or cost‐benefit analysis. Articles can be classified as both cost‐utility and cost‐benefit analyses if they contain keywords for both. Articles are disaggregated by the income group(s) of the country or countries studied. CBA: cost‐benefit analysis, CEA: cost‐effectiveness analysis, CUA: cost‐utility analysis, DALY: disability‐adjusted life‐year, QALY: quality‐adjusted life‐year

Although the term is widely (mis)used in the literature, genuine cost‐benefit analyses are very rare; we excluded many articles from our database which described themselves as CBAs of health interventions but did not value health or welfare outcomes. Of the 147 (5%) articles in our database which described themselves as CBAs, some do not in fact place a monetary value on health outcomes and should probably be described as CEAs or CUAs; however, for consistency and feasibility, our analysis of evaluation type is based on key term searches, and therefore reflect the authors' classification (Table S4). Cost‐utility analyses accounted for at least half of economic evaluations across all income levels, ranging from 50% (*n* = 52) in LICs to 62% (*n* = 1448) in HICs. The proportion of CUAs employing DALYs decreases from 87% (*n* = 45) in LICs to 2% (*n* = 35) in HICs, while the proportion employing QALYs increases from 13% (*n* = 7) in LICs to 35% (*n* = 23) in lower‐MICs, 68% (*n* = 123) in upper‐MICs, and 96% (*n* = 1385) in HICs. A very small proportion of studies described themselves as CUAs but did not contain any search terms for DALYs or QALYs (Figure 4 and Table S9).

### 3.6 Authors' geographical and institutional affiliations

Author affiliation data were obtained for all articles. At least one author was affiliated with an institution in the USA or the UK on 1145 (40%) and 619 (22%) of articles respectively (Table 5). China‐based authors contributed to 4% (*n* = 116) of all articles, making it the ninth largest contributor to economic evaluations, while Brazil (51, 2%) and South Africa (49, 2%) also ranked within the top 20 country affiliations. With 22 articles (1%), India was the highest ranking lower MIC and ranked 29th overall, just ahead of Hong Kong and Singapore. Uganda was the largest contributor to economic evaluations amongst LICs with 20 articles (1%) and ranked 32nd overall just ahead of New Zealand. In general, the lists of leading country affiliations of authors within each income group strongly resemble the leading countries studied. Even for Uganda, however, the largest LIC contributor, 30 of the 49 articles about the country did not include any Uganda‐based authors; of these, 25 were studies set in at least 15 countries each, but 5 articles focused on 3 or fewer countries.

**Table 5:** Most frequent countries of institutional affiliation of authors

	High‐income		Upper‐middle‐income		Low‐income and lower‐middle‐income	
Rank	Country	N	Country	N	Country	N
1	USA	1145	China	116	India	22
2	UK	619	Brazil	51	Uganda	20
3	Netherlands	267	South Africa	49	Kenya	13
4	Canada	238	Thailand	37	Vietnam	11
5	Australia	191	Colombia	32	Ghana^a^	9
6	Germany	151	Mexico	26	Zambia^a^	9
7	Spain	147	Iran	25	Nigeria	8
8	Switzerland	104	Turkey	18	Indonesia^a^	5
9	France	103	Argentina	14	Burkina Faso^a^	5
10	Italy	99	Malaysia	12	Bangladesh^a^	4
11	Sweden	98	Peru	9	Pakistan^a^	4
12	Belgium	78	Bulgaria^a^	7	Tanzania^a^	4
13	Japan	53	Serbia^a^	7	Philippines^a^	4
14	Denmark	45	Hungary	5	Egypt^a^	4
15	Ireland	39	Venezuela	3	Ethiopia^a^	2
16	Norway	32	Romania^a^	2	Malawi^a^	2
17	Taiwan	28	Lebanon^a^	2	Congo, Dem. Rep.^a^	2
18	Finland	27	Costa Rica^a^	2	Benin^a^	2
19	Korea, Rep.^a^	25	Jordan^a^	2	Myanmar^a^	2
20	Austria^a^	25	Tunisia^a^	2	Zimbabwe^a^	2
21	Greece	23	Iraq^a^	1	Cameroon^a^	2
22	Hong Kong	21	Botswana^a^	1	Senegal^a^	2
23	Singapore	21	Cuba^a^	1	Sri Lanka^a^	1
24	New Zealand^a^	19	Kazakhstan^a^	1	Cambodia^a^	1
25	Poland^a^	19	Panama^a^	1	Niger^a^	1
26	Portugal	15	Jamaica^a^	1	Afghanistan^a^	1
27	Israel	12	Dominican Republic^a^	1	Nepal^a^	1
28	Russia	9			Rwanda^a^	1
29	Chile	8			Sierra Leone^a^	1
30	Czech Republic	7			Somalia^a^	1
31	Slovenia^a^	5			Syria^a^	1
32	Qatar^a^	5			Bolivia^a^	1
33	Croatia^a^	2			Guyana^a^	1
34	Saudi Arabia^a^	2			Uzbekistan^a^	1
35	Estonia^a^	2			West Bank and Gaza^a^	1
36	Iceland^a^, Liechtenstein^a^, Lithuania^a^, Macao^a^, Malta^a^, Puerto Rico^a^, Trinidad and Tobago^a^	1				

The table ranks countries of institutional affiliations of authors by the number of economic evaluations including at least one author affiliated with that country. All countries affiliated with at least one author of at least one economic evaluation are listed.

a Equal ranking with country above and/or below.

On 91% of articles, at least one author was based in a HIC (Table 6). All but 5 of the 2350 articles studying HICs included at least one author based in a HIC and most articles studying upper‐MICs included at least one upper MIC‐based author (*n* = 338, 86%). By contrast, only 53 and 54% of articles studying LICs and lower‐MICs, respectively, included any author based in an institution in the respective income group. Authors based in upper‐MICs contributed to a relatively small proportion of articles analyzing LICs (*n* = 16, 15%) or lower‐MICs (*n* = 15, 12%), and in nearly half of these articles, upper‐MICs were also studied. Authors based in HIC institutions contributed to 94% (*n* = 98) of articles analyzing LICs and 82% (*n* = 99) analyzing lower‐MICs, compared with fewer than half of evaluations in upper‐MICs (*n* = 175, 45%). Of the 65 articles studying LIC and lower‐MIC which did not include an author from those income levels, 44 articles included at least one author based in the USA (68%). At least one author listed a major pharmaceutical company amongst the institutional affiliations on 9% of articles (*n* = 246) overall, varying from 9% (*n* = 221) of articles studying HICs, to 12% (*n* = 46) studying an upper‐MIC, 7% (*n* = 8) studying a lower‐MIC and 4% (*n* = 4) studying a LIC. English is an official language in four of the top five HICs and LICs and lower‐MICs contributing to economic evaluations, compared with just one of the top five upper‐MICs (Table 7).

**Table 6:** Income group studied versus income group of author affiliations

	Income group of authors' country affiliation(s)									
Income group of countries studied	Low		Lower‐middle		Upper‐middle		High		Total	
Low	55	(53%)	7	(7%)	16	(15%)	98	(94%)	104	(100%)
Lower‐middle	8	(7%)	65	(54%)	15	(12%)	99	(82%)	121	(100%)
Upper‐middle	11	(3%)	11	(3%)	338	(86%)	175	(45%)	391	(100%)
High	4	(0%)	12	(1%)	51	(2%)	2345	(100%)	2350	(100%)
Total	59	(2%)	80	(3%)	394	(14%)	2601	(91%)	2844	(100%)

Row percentages are presented and reflect the proportion of articles addressing a given income level, which include authors affiliated with institutions based in a country of the given income level.

**Table 7:** Most frequent institutional affiliation of authors

Income group of authors' institutions									
	High			Upper‐middle			Low and lower‐middle		
Rank	Institution	Country	N	Institution	Country	N	Institution	Country	N
1	Harvard University	USA	152	University of Cape Town	South Africa	19	Makerere University	Uganda	14
2	Johns Hopkins University	USA	74	Tehran University of Medical Sciences	Iran	17	Kenya Medical Research Institute	Kenya	9
3	London School of Hygiene and Tropical Medicine	UK	70	Shanghai Jiao Tong University^a^	China	15	Ministry of Health	Vietnam	6
4	University of Toronto	Canada	65	Universidade de Sao Paulo^a^	Brazil	15	All India Institute of Medical Sciences^a^	India	5
5	University of Amsterdam	Netherlands	62	University of the Witwatersrand^a^	South Africa	15	Hanoi Medical University^a^	Vietnam	5
6	University College London	UK	61	Chinese Center for Disease Control and Prevention^a^	China	11	Ghana Health Service^a^	Ghana	4
7	University of York	UK	57	Mahidol University^a^	Thailand	11	Ministry of Health^a^	Zambia	4
8	Pfizer, inc.	Multinational private company	51	Instituto Mexicano del Seguro Social^a^	Mexico	10	University of Nigeria^a^	Nigeria	4
9	Centers for Disease Control and Prevention^a^	USA	46	Universidad Nacional de Colombia^a^	Colombia	10	Centre Muraz^a^	Burkina Faso	3
10	Duke University^a^	USA	46	Health Intervention and Technology Assessment Program	Thailand	8	Family Health^a^ International	Vietnam	3
							INDEPTH Network^a^	Ghana	3
							Kenya Government Medical Research Center^a^	Kenya	3
							Mbarara University of Science and Technology^a^	Uganda	3
							Ministry of Health^a^	Kenya	3
							Universitas Padjadjaran^a^	Indonesia	3
							University of Ghana^a^	Ghana	3
							YR Gaitonde Centre for AIDS Research and Education^a^	India	3

The table ranks institutional affiliations of authors by the number of economic evaluations including at least one author affiliated with that institution. The top 10 institutions located in each income level are listed. To the extent possible, institutions' totals include their affiliated hospitals, centres, and groups even if the parent institution was not specifically cited in the affiliation data.

a Equal ranking with country above and/or below.

Harvard University, including its affiliated hospitals, was by some distance the institution contributing to the largest number of economic evaluations (*n* = 152). The top institutions producing economic evaluations in LICs and lower‐MICs are notable for their low individual and collective output, as well as for including many ministries of health or (semi‐)autonomous research institutes (Table 7). The leading LIC or lower MIC institution, Makerere University, was listed amongst the author affiliations of 14 economic evaluations over the 2.3 years we studied. The WHO was listed amongst the author affiliations on 25 articles, while the World Bank and United Nations' Children's Fund contributed to only four economic evaluations each.

## 4 Discussion

Our analysis provides an evidence base from which to discuss the current state of the economic evaluation field and has generated many questions which warrant further investigation. Some of these issues are examined in other papers in this special issue. For example, Griffiths *et al*. (2016) compare the methods used in economic evaluations in countries of differing income groups in a representative sample of articles from the database we created, while other authors examine costing methods (Sweeney *et al*., 2016, Cunnama *et al*., 2016), outcome metrics (Greco *et al*., 2016), and issues around capacity to produce and to use economic evaluations (Kaló *et al*., 2016). Our analysis also offers insights to strengthen the process of prioritising, conducting, publishing, and developing capacity for economic evaluation research. Here, we discuss the state of the field and the implications of our findings for research priority setting and capacity development.

### 4.1 The state of health economic evaluation

We identified a large volume of economic evaluations—2844 over 28 months—including 1273 in 2013 alone. The principal economics database, EconLit, contains 5483 publications with ‘Health’ JEL codes for 2012 and 2013, but captured just 1% of economic evaluations published in those years. A large majority of economic evaluations were published in biomedical journals and even many of the journals we categorised as ‘health economics, services, and policy’ are not indexed in EconLit. Adding the 2413 economic evaluations we identified for 2012 and 2013 to the EconLit health records would increase the volume of ‘health economics’ research by 44%. Further, these publications still do not include the many other health economic analyses of, for example equity, demand, markets, and incentives, which are published in journals outside the economics literature as defined by the EconLit database.

Despite important analytical differences and the lack of overlap between the body of literature addressed in our analysis and Wagstaff and Culyer's analysis of health economics within the EconLit database, our findings share some commonalities. Both our analyses (along with Greenberg *et al*. (2010)) identified Harvard as the leading institution and the USA as by far the most prolific contributor to health economic (evaluation) research, followed by the UK, and then the Netherlands, Canada, and Australia. China and South Africa also rank highly in both our analyses. Nonetheless, our findings also differ in important ways. As expected, our lists of leading journals share very little in common, as economic evaluations are predominantly published in biomedical journals, which are not indexed in EconLit. Some contributors, such as the World Bank and Taiwan, which ranked very highly in Wagstaff and Culyer's analysis, contribute far less to economic evaluations, while institutions with a stronger focus on health (rather than only economics) tend to rank more highly in our analysis. There are also substantial differences with respect to our estimates of the volume of research. Whereas Wagstaff and Culyer find that ‘economic evaluation . . .[shows] no clear trend’, our analysis has highlighted the substantial size of the applied health economic evaluation literature relative to the health economics literature within EconLit and indicates that with just 1% of the applied economic evaluation literature, the EconLit database is unlikely to provide a representative indication of trends over time in the size or relative importance of health economic evaluation.

As previously highlighted (Wagstaff and Culyer, 2012), identifying health economic literature in the biomedical databases was not straightforward. We found the use of economic vocabulary and article classifications in biomedical journals and databases to be so poor and inconsistent as to render simultaneously sensitive and specific searching impossible (Text S1). The NHS EED database, while incomplete, was by far the most sensitive and specific source of economic evaluations, which makes the decision to cease to update it from March 2015 particularly lamentable. The ongoing work to add DALY‐based cost‐utility analyses to the existing QALY‐based Tufts Economic Evaluation Registry is a welcome development; however, it will still omit half of economic evaluations conducted in LMICs and currently charges for access.

Our findings paint a picture of a research community that is simultaneously highly concentrated in a few countries and institutions and highly fragmented. A very small number of journals publish economic evaluations from both high‐income and low‐income settings and a large proportion of articles appear in journals which only very rarely publish economic evaluations. The fact that so many biomedical journals now publish economic evaluations (if only rarely) is a positive sign of the acceptance and integration of economic evaluation within health research. It is also perhaps unsurprising, as economic evaluations are usually oriented towards health sector decision makers. This fragmentation may, however, also explain some of the problems of quality highlighted elsewhere (Griffiths *et al*., 2016), as biomedical journal editors may not only lack specialist knowledge of economic evaluation methods but also lack familiarity with pools of suitably qualified reviewers. In this way, the small number of journals publishing economic evaluations about LMICs may present an opportunity to engage with the editors of these journals to help improve standards where necessary, whereas the vast array of authors, institutions, and journals associated with economic evaluations set in HICs presents a greater challenge. In any case, the lack of scholarly dialogue between those focusing on countries of differing income levels seems likely to be detrimental to all.

We hope that recognition of the size, importance, and fundamental interdisciplinarity of health economic evaluation will lead to an evolution in research culture within the field, and also, on a practical level, to improvements in existing databases or creation of a new one that will better reflect and serve the needs of health economics researchers. Of course, authors themselves, reviewers, and editors could already do far more to facilitate the efficient identification of health economic evaluations. For example, an initial step could include ensuring that all articles include the study design in their title, as is already required by *Plos Medicine*, and that those that are not economic evaluations avoid economic terminology, such as ‘cost‐effective’ in their titles, abstracts, and keywords.

### 4.2 Research priority setting

Our findings also raise a number of questions about the health and geographical areas that are and are not prioritised for health economic evaluation. Burden of disease is not and should not be the sole determinant of the volume of economic evaluation research. It seems difficult to argue, however, that the differences between the number of economic evaluations conducted across LICs, MICs, and HICs are equitable or efficient. HICs account for 16% of the world's population, 11% of the GBD (WHO, 2014), and 83% of all economic evaluations conducted, while LICs account for 12% of the world's population, 19% of the GBD, and 4% of economic evaluations. There are 139 different LMICs (World Bank, 2015), which have very diverse epidemiological and economic characteristics, and also, in many cases, weak(er) health systems with substantial and diverse constraints on the supply and demand for health care; this diversity likely contributes to greater heterogeneity in the cost‐effectiveness of interventions and necessitates more, not less, research (Vassall *et al*., 2016). Further, the health benefits foregone by incorrect priority setting decisions may be substantially higher in low‐income settings than in high‐income settings.

One of our most surprising findings is how well the health areas studied in HICs correlate with the burden of disease in those settings. In LMICs, however, the picture is much more mixed, with many more economic evaluations conducted about health areas accounting for lower proportions of the burden of disease. There are several reasons why such discrepancies may not be inequitable or inefficient. First, the GBD estimates themselves are highly contested (Nord, 2013, Byass *et al*., 2013); intended to reflect only a very narrow definition of health, the newest disability weights used in the GBD estimates exclude wider individual or social welfare consequences (Salomon *et al*., 2012). In the case of HIV/AIDS, for example, the many and varied stakeholders could therefore conclude that it is right that HIV should be studied more than health areas accounting for a larger burden of disease because of its wider social and economic consequences or because its health consequences are only lower than other diseases because of ongoing and expensive control efforts. Second, some health areas may have a low value of additional information relative to the costs of generating the information, especially if extensive research has already been conducted in that area. Third, so little may be understood about some health problems at a clinical level that economic evaluation of interventions may be premature. Fourth, economic evaluations may be conducted not to consider adding another more effective and more costly intervention, but rather to consider divestment from costly interventions, and therefore economic evaluations in health areas that contribute very little to the disease burden may be warranted. Finally, as economic evaluations are conceptualised around a (package of) interventions, which may not map neatly onto specific conditions, categorization of economic evaluations by health areas also has some conceptual limitations, which could weaken their correlation with disease burden; we found this to be particularly true for surgical procedures, pain management and palliative care, and health systems and intersectoral interventions.

On the other hand, the four health areas accounting for the largest burden of disease in LICs are as follows: (i) neonatal and maternal conditions; (ii) respiratory diseases; (iii) wounds and injuries; and (iv) diarrhoeal diseases. While further biomedical advances, such as a point‐of‐care test for bacterial infections would help (Zumla *et al*., 2014), the bulk of the impact of all four of these health areas needs to be addressed through health systems, multi‐sectoral, and/or social interventions such as prompt access to high‐quality health facilities (Kerber *et al*., 2007), road safety measures (WHO, 2013), and improved water and sanitation (Bartram *et al*., 2005). Such solutions offer little potential for pharmaceutical company profits and instead require complex interventions. Recent systematic reviews of economic evaluations of cardiovascular disease interventions in LMICs similarly found that evaluations of pharmacological interventions dominated and a greater focus on evaluation of non‐clinical strategies were needed (Shroufi *et al*., 2013, Suhrcke *et al*., 2012). Financing such evaluations is unlikely to appeal to private for‐profit companies, and so domestic and international research funders, as well as researchers themselves, should concentrate on producing research in these areas, and thereby correct this market failure.

### 4.3 Capacity development

Several of our findings have important implications for thinking about how to increase capacity to produce and to use high‐quality and policy‐relevant health economic evaluations. Large upper‐MICs, especially China but also South Africa, Brazil, and Iran, produce substantial numbers of economic evaluations and far more than many smaller HICs. This is in some ways unsurprising, as the costs of research are independent of the size of a country's population or economy and so the relative costs of research are lower in large economies. Capacity development is important for all countries, but particularly challenging for LMICs and for small HICs as well (Kaló *et al*., 2016). A large gap between the numbers of economic evaluations conducted and what is needed for priority setting persists in all but a few countries (Geroy, 2012, Odame, 2013, Mori and Robberstad, 2012).

Our analysis has identified some clear institutional leaders in LMICs, but also highlighted that many countries produce few, if any, economic evaluations. We propose the development of strong regional or sub‐regional networks, which bring together existing capacity in health economic evaluation and build on centres of strength in health intervention research, even where substantial economic evaluation capacity may not yet exist. A multi‐stakeholder report on how to strengthen health economics more generally in Africa highlighted the importance of international networks as well as local institutional support (McIntyre *et al*., 2008). In addition to training and ongoing technical support, a well‐funded regional network could also offer scope for deeper collaboration in producing multi‐country evaluations and assessing transferability of findings across the region. Such a regional approach could be more efficient in generating economic evidence and assessing its relevance to a wider range of settings more systematically.

The leading contributors to economic evaluations from LICs and lower‐MICs tend to be research institutions, often within or associated with ministries of health, rather than universities. Such embeddedness should be an advantage in ensuring that research both reflects and informs a country's health priorities. It also means, however, that there may be no pre‐existing link between those who conduct health economic evaluation research and those who teach and train undergraduate and postgraduate students in these countries. This marked difference from HICs and even upper‐MICs may require new approaches to capacity development, rather than replication of strategies that have achieved successes in upper‐MICs and HICs.

At the same time, further work is needed to generate demand for economic evaluation both at national level, through the institutionalization of priority setting (Odame, 2013, Mori and Robberstad, 2012), and globally, through transparent priority‐setting initiatives at global funding bodies and continuing efforts to strengthen the role of economic evaluation in policy making at the WHO (Wiseman *et al.,* 2016), whose policy recommendations play a particularly large role in LICs and lower‐MICs (WHO, 2012).

Finally, nearly half of economic evaluations studying LICs and lower‐MICs do not include any authors from LMIC institutions. Some of these were desk‐based modelling studies; however, many involved data collection in LMICs. Some may have included authors from LMICs affiliated with a HIC institution, for example as doctoral students; however, such cases cannot explain the full magnitude of the discrepancy. It is unclear whether this discrepancy reflects a lack of opportunities for participation from fellow researchers or funders, lack of skills or incentives, or some combination of these and other factors, but the results are clearly inequitable (Chu *et al*., 2014). The situation also suggests a failure to recognise the wider potential of research capacity development to improve health in LMICs and the more immediate impact that real partnership with LMIC researchers and policy makers can have in ensuring that the research is policy‐relevant and informs policy decisions. Both funders and researchers in all countries must examine and address these inequities.

We hope that the findings of this analysis will be useful for those conducting (systematic) reviews of the economic evaluation literature and that they will encourage and provide an empirical grounding for debate on the current state and future directions for this growing field.

## Conflict of Interest

The authors have declared that there is no conflict of interest.

## Ethics Statement

As the analysis is based entirely on publicly available data, specific ethical approval was not required.

## Supporting information

Supporting info item
