# Supplementary material for: Economic Evaluation in Global Perspective: A Bibliometric Analysis of the Recent Literature
Source: Health Econ. 2016 Jan 25;25(Suppl Suppl 1):9–28. doi: 10.1002/hec.3305 (PMC5042080; doi:10.1002/hec.3305)
Supplement: Supplementary file 1 — Supporting info item [file HEC-25-9-s001.pdf]

SUPPLEMENTARY MATERIALS

Economic evaluation in global perspective: A bibliometric analysis of the recent literature

PITT, C., GOODMAN, C. & HANSON, K. 2016. Economic evaluation in global perspective: A bibliometric analysis of the recent literature. *Health Economics,* **25** (Suppl. S1).

Contents

SUPPLEMENTARY FIGURE
Figure S1 Flow diagram of the data development process

SUPPLEMENTARY TEXT
Text S1 A note on database indexing terms
Text S2 Supplementary information on article classification

SUPPLEMENTARY TABLES
Table S1 Searches in bibliographic databases
Table S2 Mapping of 25 disease areas onto the Global Burden of Disease (GBD), International Classification of Disease (ICD-10), and search terms used
Table S3 Classification of journal types
Table S4 Search terms to classify cost-utility and cost-benefit analyses
Table S5 Search findings by database – all articles and databases
Table S6 Search findings by database – excluding NHS EED and Wiley HEED
Table S7 Search findings by database – only articles studying low- and middle-income countries, excluding NHS EED and Wiley HEED
Table S8 Journal concentration by income group of countries studied
Table S9 Number and proportion of economic evaluations by type and income group

References

Figure S1 Flow diagram of the data development process

The figure is adapted from the flow diagram recommended in the PRISMA statement on systematic reviews (Liberati et al., 2009). The "eligibility" stage recommended by PRISMA is not used here as articles were not reviewed for quality; decisions to include records were based primarily on the record's source, title, and abstract; the full text was only screened where the title was unclear and the abstract was not available in any of the downloaded data.

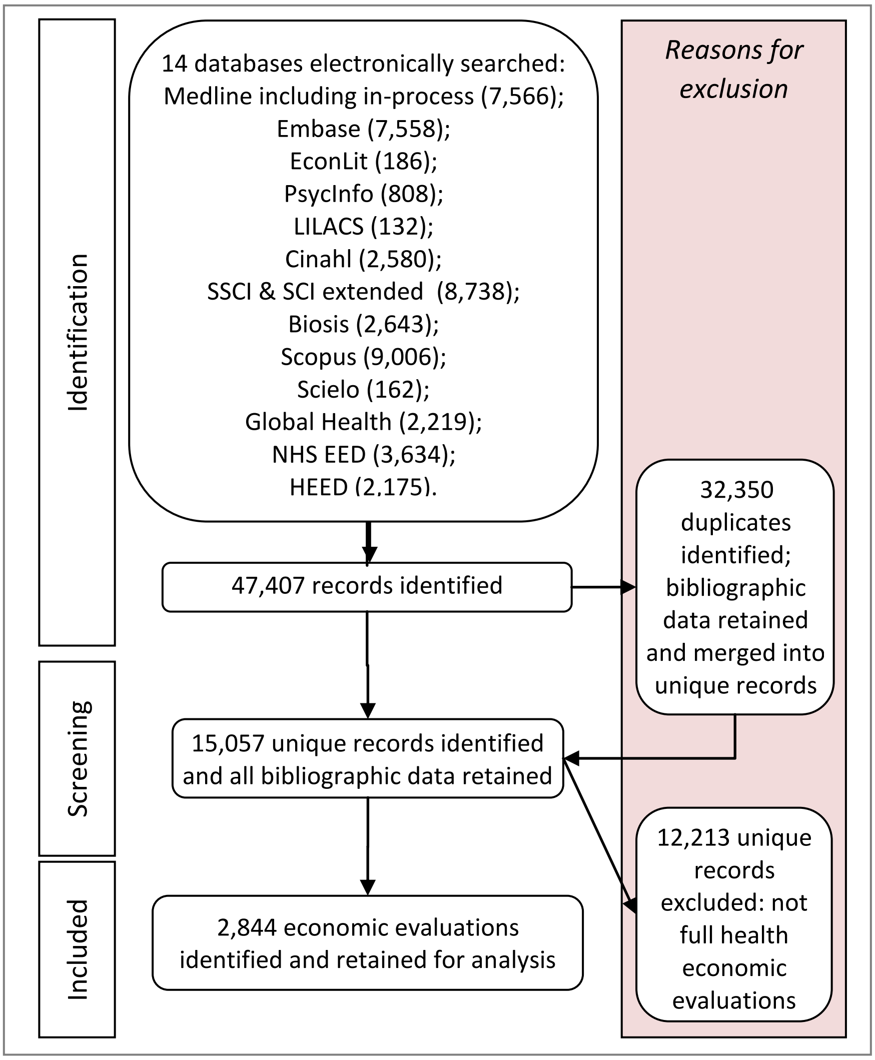

Text S1 A note on database indexing terms

In developing our search strategy, we explored the use of controlled vocabulary indexing terms, if available, in each of the databases; unlike author-defined keywords, these terms are generally applied to publications by professional indexers from a pre-defined set. While this standardization should offer advantages, one drawback is the delays in their application; while many of the databases offer basic citation data as supplied by the journal first, indexing takes more time and so searches based exclusively on indexing terms will exclude the most recent literature, to which index terms have not yet been applied.

In Medline and Embase, indexing terms are known as medical subject headings (MeSH) and Emtree (which is not an acronym), respectively; both are organized hierarchically. While the only MeSH term relevant to our search is “cost-benefit analysis”, Emtree appears much more detailed and appropriate, as it distinguishes “cost effectiveness analysis”, “cost utility analysis”, and “cost benefit analysis” from “cost control”, “cost minimization analysis”, and “cost of illness” within the broader indexing term “economic evaluation.” When we compared the results of our searches in the title, abstract, and author-defined keywords for the key terms we identified above with the results of searches using MeSH terms (in Medline) and Emtree terms (in Embase), we found that the controlled vocabulary terms were both less specific and less sensitive. Our search terms identified many relevant articles missed by the MeSH and Emtree indexers. By contrast, the controlled vocabulary terms greatly increased the number of search results, but a review of the first hundred records identified by the MeSH term and, separately, by each of the three Emtree terms (i.e. 400 records in total) after excluding records identified by our search terms identified only one additional article meeting our inclusion criteria (identified by the Emtree term “cost-effectiveness analysis”). We used this article to develop an additional set of search terms (based on “cost per x”) and concluded that the MeSH and Emtree BM indexing terms were not useful for our final searches, as they identified a vast number of articles, many of which contained no cost or other economic data or analysis , while omitting many relevant publications .

Another database applying its own indexing is HEED . On the “compound search” page, HEED offers “type of econ eval” as a search category, as well as a “type of article”. While the associated picklist does not make this obvious, HEED in fact categorizes economic evaluations as “cost effectiveness analysis”, “cost utility analysis”, “cost benefit analysis”, “cost analysis”, “cost of illness”, “cost benefit analysis”, and “cost consequences analysis”; it allows a single record to be classified as multiple types of economic evaluation, allows the user to specify only “applied study” as the “type of article”, and reports that its indexers are professional health economists. After examining this classification, we found that the terms for CEA, CUA, and CBA were highly specific and useful when combined with “applied study” as type of study , however, many publications in the HEED database were not classified at all, making the search relatively insensitive even within the HEED database. In HEED, we therefore implemented two separate searches: 1) using the HEED classification of the type of economic evaluation, and 2) using our search terms in the title, abstract, and author-defined keywords, and excluding records containing the specified categories, such that any records identified by this search would be additional to records identified by the use of HEED’s indexing.

The EconLit database uses the Journal of Economic Literature (JEL) classification system, however, unlike the indexing systems previously described, JEL codes are applied by the authors themselves. They break down the wider health economics field into 6 specified sub-fields, none of which mention in their descriptions or examples either applied or methodological work in economic evaluation; “general” and “other” health economics categories are also provided. On reviewing a

selection of health economic evaluations in the EconLit database identified by title and abstract searches, we found that while some authors combine the codes “D61: Allocative Efficiency; Cost-benefit analysis” (within the microeconomics heading) and “I12: Health Production” (within the health economics heading), other authors did not use these codes at all, choosing instead a wide variety of other codes within the health, microeconomics, and “miscellaneous” headings in particular, as well as others. Rather than using the JEL codes, we therefore decided to take a more sensitive approach in EconLit, and instead searched for “health” in all fields, which would capture the word “health” in JEL codes, but also in journal title, keywords, article title, or abstract; we combined this with keyword searches for our definition of economic evaluation.

Text S2 Supplementary information on article classification

Health areas

We developed a classification of 25 health areas so as to allow comparability with the Global Burden of Disease (GBD) estimates (World Health Organization., 2014), to be implementable with an electronic key term search, and to permit meaningful analysis. The GBD uses four hierarchical levels to classify disease. At its highest level, it classifies diseases as “Communicable, maternal, perinatal and nutritional conditions”, “Non-communicable diseases” or “Injuries”, while at its lowest levels, it breaks these down into 154 more specific conditions. We did not maintain the GBD’s highest level classification because in some cases, it was not implementable (e.g. key term searches could not distinguish between communicable and non-communicable causes of respiratory diseases) and in other cases, we felt the distinction did not map coherently onto preventive and curative interventions (e.g. we separated “intentional injuries: self harm” from other injury categories and placed it in a single category with mental health issues).

A set of up to 49 search terms was developed for each of our health areas through an iterative process. We began by reviewing the names of sub-categories in the GBD and the categories and descriptions provided in the ICD-10 (World Health Organization., 2011) to develop an initial set of search terms. We then reviewed the titles and keywords of unclassified records in our database, and continued adding search terms until all records in our database which could be classified were classified according to at least one health area. Throughout the process, we reviewed samples of records within each health area, and reviewed in-depth the records identified by search terms we considered potentially ambiguous, before finalizing our search terms and disease classification.

Institutional and geographic affiliations of authors

We analyzed data on the institutional affiliation of all authors to develop a comprehensive picture of the institutions and countries contributing to health economic evaluations. We began by transferring the institutional affiliation data from wide to long form and implementing the country keyword searches previously developed. As affiliation data frequently did not name a country, unclassified affiliations were then iteratively reviewed and search terms for city names and non-geographic institution names (e.g. Harvard, Yale) were identified and linked to countries, taking care to avoid misclassifying search terms such as “York”, which could refer to the city (York) or county (Yorkshire) in the United Kingdom, to York University in Canada, or to the city or state of New York in the United States. In this way, nearly all articles for which affiliation data were available were classified as being produced by researchers in one or more specified countries. This data was further cross-checked against the data on countries studied and inconsistencies reviewed. The original articles were sought to resolve inconsistencies and to obtain institutional affiliation data for any articles remaining without data. Articles were then classified by the income group of the country or countries of the author affiliations and the countries producing the greatest volume of economic evaluations were ranked within each income group.

We further identified the top ten institutions within each income group by volume of economic evaluations produced. The affiliation data for top-ranked countries within each income group were carefully reviewed to develop sets of specific key terms for institutions. As in previous work (Wagstaff and Culyer, 2012, Rubin and Chang, 2003), schools, colleges and institutes were aggregated with the university to which they belonged, with the exception of the highly federal Universities of London, California, Texas, and other similar university systems, whose constituent members were analyzed separately. To the extent possible, hospitals and institutes were associated

with their parent institution, even when that institution was not explicitly named. Even though they are independently owned and managed, Harvard's 16 affiliated hospitals were aggregated with Harvard. Once an initial set of ten institutions were identified for each income group, only affiliations from countries which had produced more than the tenth-ranked institution for that income group were reviewed to identify institutions which could have produced more economic evaluations than the currently tenth-ranked institution. For example, the tenth-ranked UMIC institution, the *Instituto Mexicano del Seguro Social*, produced 7 economic evaluations, and so only affiliations from UMICs which had produced at least 7 economic evaluations were reviewed to identify individual institutions which could have produced at least this number. The searches for city names were then used to facilitate the identification of institutions.

In addition, search terms were developed for international and inter-governmental organizations, such as United Nations agencies and the World Bank, and for multi-national pharmaceutical companies, regardless of the country, if any, with which they were associated in their affiliation data. These were then aggregated into two groups, "international organizations" and "pharmaceutical industry", to permit consideration of their relative influence.

This process allowed a comprehensive assessment of the total volume of articles produced by each country and by income group, as well as a comprehensive assessment of top institutions, taking into account the many and unpredictable variations in their listing. Less thorough approaches would be likely to bias rankings towards institutions such as Yale, with its unique name which also appears in the name of all its constituent schools and hospital, and away from institutions with a wider variety of permutations, abbreviations and possibly ambiguous versions of its name, such as the University of York (Univ York, U York, but not York University), with Hull-York Hospital (Hull-York Hosp), which were not always listed with the university name in the affiliation data.

We considered a number of possible approaches for analysing articles with more than one institutional affiliation. Both Wagstaff and Culyer (2012) and Rubin and Chang (2003) were constrained by the EconLit database, which only provides data on the first three or four authors, whereas we obtained institutional affiliation data for all authors. We considered assigning a fractional value (and even weighted fractional values reflecting author order) to each institution based on the number of different authors or institutions represented on a given article (Aksnes et al., 2012, Hagen, 2013, Retzer and Jurasinski, 2009). However, we rejected such approaches for two reasons: first, we believe that the use of zero-sum metrics establishes a perverse incentive against collaboration between institutions and against the crediting of collaborators. We therefore assigned one point per institution per article, regardless of the number of institutions or authors on a given article. This has the disadvantage of weighting the analysis towards articles from multiple institutions, as these articles are counted multiple times.

Table S1 Searches in bibliographic databases

Database (Interface)	Search
**National Health Service Economic Evaluation Database (Centre for Reviews and Dissemination)**<br><br>***Note: this database is still available, however, updating ceased in March 2015***	NHS EED: tick box (DARE: blank, HTA: blank)<br>Publication year: 2012 to 2014<br><br>Export: “Full record” → produces a single RIS document with consecutively numbered records
**Health Economic Evaluations `Database (Wiley)**<br><br>***Note: this database ceased to be available from the end of 2014.***	**Search 1: Identifies studies already reviewed and categorized by HEED**<br>Search type (from purple button on left): Compound Search<br>Use pull-down menus to select:<br>| Journal Date | >= 2012 | AND | |Type of Econ eval | 'EFFECTIVENESS' Or 'UTILITY' Or 'BENEFIT' | AND | | Type of Article | ‘APPLIED’ | <br>**Search 2: Identifies additional studies not yet reviewed and categorized by HEED**<br>Search type (from purple button on left): Expert Search<br><br>1. EE= 'EFFECTIVENESS' Or 'UTILITY' Or 'BENEFIT' AND TE= 'APPLIED' AND JD>= 2012<br>2. TI='Cost-effective*' OR 'Cost-Utility' OR 'Cost-benefit' OR 'Cost effective*' OR 'Cost Utility' OR 'Cost benefit' OR 'economic evaluation' AND (JD>= 2012)<br>3. AB='Cost-effectiveness' OR 'Cost-Utility' OR 'Cost-benefit' OR 'Cost effectiveness' OR 'Cost Utility' OR 'Cost benefit' OR 'economic evaluation' AND (JD>= 2012)<br>4. KW='Cost-effectiveness' OR 'Cost-Utility' OR 'Cost-benefit' OR 'Cost effectiveness' OR 'Cost Utility' OR 'Cost benefit' OR 'economic evaluation' AND (JD>= 2012)<br>5. AB='cost per death averted' or 'cost per death avoided' or 'cost per case averted' or 'cost per case avoided' or 'cost per infection' or 'cost per life' or 'cost per disability-adjusted' or 'cost per quality-adjusted' or 'cost per qaly' or 'cost per daly'<br>6. CS = LINE 1 OR LINE 2 OR LINE 3 OR LINE 5
**LILACS (Bireme/WHO/ PAHO)**	cost-effective or "cost effective" or cost-effectiveness or "cost effectiveness" or cost-utility or "cost utility" or cost-benefit or "cost benefit" or "economic evaluation" or "cost per death" or "cost per case" or "cost per infection" or "cost per life" or "cost per disability adjusted" or "cost per quality adjusted" or "cost per disability-adjusted" or "cost per quality-adjusted" or "cost per qaly" or "cost per

	daly" [Words]<br>and<br>2012 or 2013 or 2014 [Country, year publication]<br>and<br>not study and protocol [Title words]
**ADOLEC (Bireme/WHO/PAHO)**	cost-effective or "cost effective" or cost-effectiveness or "cost effectiveness" or cost-utility or "cost utility" or cost-benefit or "cost benefit" or "economic evaluation" or "cost per death" or "cost per case" or "cost per infection" or "cost per life" or "cost per disability adjusted" or "cost per quality adjusted" or "cost per disability-adjusted" or "cost per quality-adjusted" or "cost per qaly" or "cost per daly" [Words]<br>and<br>2012 or 2013 or 2014 [Country, year publication]<br>and not<br>study and protocol [Title words]
**Medline (including in-process) (Ovid SP)**	1. ("2012" or "2013" or "2014").yr.<br>2. (cost-effectiveness or cost-utility or cost-benefit or "economic evaluation").ti,ab,kw.<br>3. cost-effective.ti.<br>4. ("cost-per-death-av*" or "cost-per-case-av*" or "cost-per-infection" or "cost-per-life" or "cost-per-disability-adjusted-life-year" or "cost-per-quality-adjusted-life-year" or "cost-per-qaly" or "cost-per-daly").ti,ab,kw.<br>5. 2 or 3 or 4<br>6. 1 and 5<br>7. limit 6 to (autobiography or bibliography or biography or case reports or classical article or comment or congresses or consensus development conference or consensus development conference, nih or editorial or festschrift or guideline or historical article or in vitro or interactive tutorial or interview or lectures or letter or news or newspaper article or patient education handout or practice guideline or published erratum or technical report or twin study or video-audio media or webcasts)<br>8. 6 not 7<br>9. limit 8 to animals<br>10. limit 9 to human<br>11. 9 not 10<br>12. 8 not 11<br>13. study protocol.ti.<br>14. 12 not 13
**Embase (Ovid SP)**	1. ("2012" or "2013" or "2014").yr.<br>2. (cost-effectiveness or cost-utility or cost-benefit or "economic evaluation").ti,ab,kw.<br>3. cost-effective.ti.<br>4. ("cost-per-death-av*" or "cost-per-case-av*" or "cost-per-infection" or "cost-per-life" or "cost-per-disability-adjusted-life-year" or "cost-per-quality-adjusted-life-year" or "cost-per-qaly" or "cost-per-daly").ti,ab,kw.<br>5. 2 or 3 or 4<br>6. 1 and 5<br>7. limit 6 to (book or book series or conference abstract or conference paper or conference proceeding or "conference review" or

	editorial or erratum or letter or note or report)<br>8. 6 not 7<br>9. limit 8 to (animals or animal studies)<br>10. limit 9 to humans<br>11. 9 not 10<br>12. 8 not 11<br>13. study protocol.ti.<br>14. 12 not 13
**EconLit (Ovid SP)**	1. ("2012" or "2013" or "2014").yr.<br>2. health.af.<br>3. (cost-effective* or cost-utility or cost-benefit or "economic evaluation").af.<br>4. ("cost-per-death-av*" or "cost-per-case-av*" or "cost-per-infection" or "cost-per-life" or "cost-per-disability-adjusted-life-year" or "cost-per-quality-adjusted-life-year" or "cost-per-qaly" or "cost-per-daly").af.<br>5. 3 or 4<br>6. 1 and 2 and 5<br>7. study protocol.ti.<br>8. limit 6 to (books or book reviews or collective volume articles or dissertations)<br>9. 6 not 8<br>10. limit 9 to working papers<br>11. 9 not 10
**PsycInfo(Ovid SP)**	1. ("2012" or "2013" or "2014").yr.<br>2. (cost-effectiveness or cost-utility or cost-benefit or "economic evaluation").mp. [mp=title, abstract, heading word, table of contents, key concepts, original title, tests & measures]<br>3. cost-effective.ti.<br>4. ("cost-per-death-av*" or "cost-per-case-av*" or "cost-per-infection" or "cost-per-life" or "cost-per-disability-adjusted-life-year" or "cost-per-quality-adjusted-life-year" or "cost-per-qaly" or "cost-per-daly").ti,ab,kw.<br>5. 2 or 3 or 4<br>6. 1 and 5<br>7. limit 6 to ("0200 book" or "0240 authored book" or "0280 edited book" or "0300 encyclopedia" or "0400 dissertation abstract" or "column/opinion" or "comment/reply" or dissertation or editorial or encyclopedia entry or "erratum/correction" or letter or obituary)<br>8. 6 not 7<br>9. limit 8 to animal<br>10. limit 9 to human<br>11. 9 not 10<br>12. 8 not 11<br>13. study protocol.ti.<br>14. 12 not 13
**Global Health (Ovid SP)**	1. ("2012" or "2013" or "2014").yr.<br>2. (cost-effectiveness or cost-utility or cost-benefit or "economic evaluation").af.

	3. cost-effective.ti.<br>4. ("cost-per-death-av*" or "cost-per-case-av*" or "cost-per-infection" or "cost-per-life" or "cost-per-disability-adjusted-life-year" or "cost-per-quality-adjusted-life-year" or "cost-per-qaly" or "cost-per-daly").af.<br>5. 2 or 3 or 4<br>6. 1 and 5<br>7. limit 6 to (annual report or annual report section or book or book chapter or bulletin or conference or conference proceedings or conference paper or correspondence or editorial or patent or thesis)<br>8. 6 not 7<br>9. study protocol.ti.<br>10. 8 not 9
**Scopus (Scopus)**	**MAIN SEARCH:**<br>((((TITLE("cost-effective*" OR "cost-utility" OR "cost-benefit" OR "economic evaluation") AND SUBJAREA(mult OR agri OR bioc OR immu OR neur OR phar OR mult OR medi OR nurs OR vete OR dent OR heal OR mult OR arts OR busi OR deci OR econ OR psyc OR soci) AND PUBYEAR > 2011) OR (TITLE("cost per death" OR "cost per case" OR "cost per infection" OR "cost per life" OR "cost per disability-adjusted" OR "cost per quality-adjusted" OR "cost per qaly" OR "cost per daly") AND SUBJAREA(mult OR agri OR bioc OR immu OR neur OR phar OR mult OR medi OR nurs OR vete OR dent OR heal OR mult OR arts OR busi OR deci OR econ OR psyc OR soci) AND PUBYEAR > 2011) OR (ABS("cost-effectiveness" OR "cost-utility" OR "cost-benefit" OR "economic evaluation") AND SUBJAREA(mult OR agri OR bioc OR immu OR neur OR phar OR mult OR medi OR nurs OR vete OR dent OR heal OR mult OR arts OR busi OR deci OR econ OR psyc OR soci) AND PUBYEAR > 2011) OR (ABS("cost per death" OR "cost per case" OR "cost per infection" OR "cost per life" OR "cost per disability-adjusted" OR "cost per quality-adjusted" OR "cost per qaly" OR "cost per daly") AND SUBJAREA(mult OR agri OR bioc OR immu OR neur OR phar OR mult OR medi OR nurs OR vete OR dent OR heal OR mult OR arts OR busi OR deci OR econ OR psyc OR soci) AND PUBYEAR > 2011) OR (AUTHKEY("cost-effectiveness" OR "cost-utility" OR "cost-benefit" OR "economic evaluation") AND SUBJAREA(mult OR agri OR bioc OR immu OR neur OR phar OR mult OR medi OR nurs OR vete OR dent OR heal OR mult OR arts OR busi OR deci OR econ OR psyc OR soci) AND PUBYEAR > 2011)) AND (SUBJAREA(mult OR immu OR neur OR phar OR mult OR medi OR nurs OR dent OR heal OR deci OR econ OR psyc))) AND NOT (TITLE("study protocol"))) AND NOT (DOCTYPE(bk OR ch OR bz OR cp OR cr OR ed OR er OR le OR no OR pr OR rp OR sh))<br><br>**BREAKING UP THE SEARCH:**<br>**BY YEAR: 2014 – 1,216**<br><br>**BY YEAR: 2013 – 4,039**<br>**Broke this one up further – by "cost-effectiveness" in title, abstract, and keywords (3,148) and not (891)**<br>**BY YEAR: 2012 – 3,751**
**Social Science Citation Index & Science Citation Index extended (Web of Science)**	# 1 4,006,203 PY=(2012 or 2013 or 2014)<br># 2 5,706 TI=("cost-effective*" or "cost-benefit" or "cost-utility" or "economic evaluation" or "cost per death" or "cost per case" or "cost per infection" or "cost per life" or "cost per disability-adjusted" or "cost per quality-adjusted" or "cost per qaly" or "cost per daly")<br># 3 13,274 TS=("cost-effectiveness" or "cost-benefit" or "cost-utility" or "economic evaluation" or "cost per death" or "cost per case" or "cost per infection" or "cost per life" or "cost per disability-adjusted" or "cost per quality-adjusted" or "cost per qaly" or "cost

	per daly")<br># 4 1,091 TITLE: ("study protocol")<br># 5 14,237 #3 OR #2<br># 6 14,237 #5 AND #1<br># 7 14,054 #6 not #4<br># 8 2,900 (#7) AND DOCUMENT TYPES: (Abstract of Published Item OR Art Exhibit Review OR Bibliography OR Biographical-Item OR Book OR Book Chapter OR Book Review OR Chronology OR Correction OR Correction, Addition OR Dance Performance Review OR Database Review OR Discussion OR Editorial Material OR Excerpt OR Fiction, Creative Prose OR Film Review OR Hardware Review OR Item About an Individual OR Letter OR Meeting Abstract OR Meeting Summary OR Music Performance Review OR Music Score OR Music Score Review OR News Item OR Note OR Poetry OR Proceedings Paper OR Record Review OR Reprint OR Script OR Software Review OR TV Review, Radio Review OR TV Review, Radio Review, Video OR Theater Review)<br># 9 11,154 #7 NOT #8<br># 10 8,738 #9 AND WC=(PATHOLOGY OR BEHAVIORAL SCIENCES OR MEDICAL LABORATORY TECHNOLOGY OR BIOLOGY OR SOCIAL SCIENCES INTERDISCIPLINARY OR SOCIAL WORK OR HEALTH CARE SCIENCES SERVICES OR VIROLOGY OR MEDICINE GENERAL INTERNAL OR HEALTH POLICY SERVICES OR PUBLIC ENVIRONMENTAL OCCUPATIONAL HEALTH SCI OR PUBLIC ENVIRONMENTAL OCCUPATIONAL HEALTH OR MICROBIOLOGY OR PHARMACOLOGY PHARMACY OR RHEUMATOLOGY OR SOCIAL SCIENCES BM OR ECONOMICS OR NEUROSCIENCES OR PARASITOLOGY OR SURGERY OR ONCOLOGY OR REHABILITATION OR DENTISTRY ORAL SURGERY MEDICINE OR OPERATIONS RESEARCH MANAGEMENT SCIENCE OR FOOD SCIENCE TECHNOLOGY OR PUBLIC ENVIRONMENTAL OCCUPATIONAL HEALTH SSCI OR CARDIAC CARDIOVASCULAR SYSTEMS OR CRITICAL CARE MEDICINE OR INFECTIOUS DISEASES OR NURSING OR IMMUNOLOGY OR OPHTHALMOLOGY OR ANESTHESIOLOGY OR CLINICAL NEUROLOGY OR EMERGENCY MEDICINE OR MULTIDISCIPLINARY SCIENCES OR NUTRITION DIETETICS OR TOXICOLOGY OR MEDICINE RESEARCH EXPERIMENTAL OR SUBSTANCE ABUSE OR PSYCHIATRY OR DERMATOLOGY OR GASTROENTEROLOGY HEPATOLOGY OR GENETICS HEREDITY OR TRANSPLANTATION OR OBSTETRICS GYNECOLOGY OR TROPICAL MEDICINE OR MEDICAL INFORMATICS OR GERIATRICS GERONTOLOGY OR OTORHINOLARYNGOLOGY OR PERIPHERAL VASCULAR DISEASE OR ORTHOPEDICS OR MANAGEMENT OR PEDIATRICS OR UROLOGY NEPHROLOGY OR PSYCHIATRY SCI OR RESPIRATORY SYSTEM OR ENDOCRINOLOGY METABOLISM OR RADIOLOGY NUCLEAR MEDICINE MEDICAL IMAGING OR PRIMARY HEALTH CARE OR ENGINEERING ENVIRONMENTAL OR HEMATOLOGY) Indexes=SCI-EXPANDED, SSCI Timespan=2012-2014
**Scielo (Web of Science)**	# 1 79,842 PY=(2012 or 2013 or 2014)<br># 2 98 TI= ("cost-effective*" or "cost-benefit" or "cost-utility" or "economic evaluation" or "cost per death" or "cost per case" or "cost per infection" or "cost per life" or "cost per disability-adjusted" or "cost per quality-adjusted" or "cost per qaly" or "cost per daly")<br># 3 221 TS=("cost-effectiveness" or "cost-benefit" or "cost-utility" or "economic evaluation" or "cost per death" or "cost per case" or "cost per infection" or "cost per life" or "cost per disability-adjusted" or "cost per quality-adjusted" or "cost per qaly" or "cost per daly")<br># 4 227 #2 or #3<br># 5 2 TI=("study protocol")<br># 6 227 #4 not #5

	# 7 175 #6 AND SU=(PUBLIC ENVIRONMENTAL OCCUPATIONAL HEALTH OR LEGAL MEDICINE OR GENERAL INTERNAL MEDICINE OR PEDIATRICS OR CARDIOVASCULAR SYSTEM CARDIOLOGY OR DENTISTRY ORAL SURGERY MEDICINE OR ANESTHESIOLOGY OR DERMATOLOGY OR PHARMACOLOGY PHARMACY OR ENDOCRINOLOGY METABOLISM OR GASTROENTEROLOGY HEPATOLOGY OR ENVIRONMENTAL SCIENCES ECOLOGY OR HEALTH CARE SCIENCES SERVICES OR NURSING OR MEDICAL LABORATORY TECHNOLOGY OR NEUROSCIENCES NEUROLOGY OR OPERATIONS RESEARCH MANAGEMENT SCIENCE OR OTORHINOLARYNGOLOGY OR OBSTETRICS GYNECOLOGY OR OPHTHALMOLOGY OR PSYCHIATRY OR PATHOLOGY OR RESPIRATORY SYSTEM OR RADIOLOGY NUCLEAR MEDICINE MEDICAL IMAGING OR TROPICAL MEDICINE OR SURGERY OR RHEUMATOLOGY OR UROLOGY NEPHROLOGY OR BIOTECHNOLOGY APPLIED MICROBIOLOGY OR INFECTIOUS DISEASES OR EConomics)<br># 8 13 (#7) AND DOCUMENT TYPES: (Announcement OR Article-Commentary OR Case-Report OR Editorial OR Letter OR Rapid-Communication)<br># 9 162 #7 not #8
**Biosis (Web of Science)**	# 1 1,608,659 PY=(2012 or 2013 or 2014)<br># 2 1,741 TI= ("cost-effective*" or "cost-benefit" or "cost-utility" or "economic evaluation" or "cost per death" or "cost per case" or "cost per infection" or "cost per life" or "cost per disability-adjusted" or "cost per quality-adjusted" or "cost per qaly" or "cost per daly")<br># 3 3,846 TS=("cost-effectiveness" or "cost-benefit" or "cost-utility" or "economic evaluation" or "cost per death" or "cost per case" or "cost per infection" or "cost per life" or "cost per disability-adjusted" or "cost per quality-adjusted" or "cost per qaly" or "cost per daly")<br># 4 4,109 #2 or #3<br># 5 4,109 #1 and #4<br># 6 35 TI= ("study protocol")<br># 7 4,104 #5 not #6<br># 8 777 (#7) AND DOCUMENT TYPES: (Annual Report OR Article Thesis Dissertation OR Book OR Book Chapter OR Book Review OR Company Profile OR Index OR Letter OR Main Cite OR Meeting OR Meeting Paper OR Obituary OR Patent OR Reprint OR Software OR Technical Report OR Thesis Dissertation)<br># 9 3,327 #7 NOT #8<br># 10 123 (#9) AND LITERATURE TYPE: (Annual Report OR Bibliography OR Biography OR Catalog OR Checklist OR Correction OR Dictionary OR Editorial OR Errata OR Identification Guide OR Manual OR Meeting Abstract OR Meeting Address OR Meeting Paper OR Meeting Poster OR Meeting Report OR Meeting Slide OR Meeting Summary OR Nomenclator OR Nomenclature OR Obituary OR Protocol OR Retraction OR Software Review OR Standard OR Taxonomic Key OR Taxonomic Review)<br># 11 3,204 #9 not #10<br># 12 2,643 #11 AND SU=(TOXICOLOGY OR RADIOLOGY NUCLEAR MEDICINE MEDICAL IMAGING OR REPRODUCTIVE BIOLOGY OR HEMATOLOGY OR ANESTHESIOLOGY OR DEVELOPMENTAL BIOLOGY OR INFECTIOUS DISEASES OR ORTHOPEDICS OR PUBLIC ENVIRONMENTAL OCCUPATIONAL HEALTH OR BEHAVIORAL SCIENCES OR EVOLUTIONARY BIOLOGY OR IMMUNOLOGY OR REHABILITATION OR ONCOLOGY OR NURSING OR PHYSIOLOGY OR CARDIOVASCULAR SYSTEM CARDIOLOGY OR NUTRITION DIETETICS OR DENTISTRY ORAL SURGERY MEDICINE OR NEUROSCIENCES NEUROLOGY OR ALLERGY OR SURGERY OR OPHTHALMOLOGY OR GENETICS HEREDITY OR OBSTETRICS GYNECOLOGY OR GASTROENTEROLOGY HEPATOLOGY OR DERMATOLOGY OR GENERAL INTERNAL MEDICINE OR

	PARASITOLOGY OR HEALTH CARE SCIENCES SERVICES OR PSYCHIATRY OR PEDIATRICS OR GERIATRICS GERONTOLOGY OR ENDOCRINOLOGY METABOLISM OR RHEUMATOLOGY OR UROLOGY NEPHROLOGY OR RESPIRATORY SYSTEM OR OTORHINOLARYNGOLOGY)
**Cinahl (EBSCO)**	S1. TI ("cost-effective*" or "cost-benefit" or "cost-utility" or "economic evaluation" or "cost per death" or "cost per case" or "cost per infection" or "cost per life" or "cost per disability-adjusted" or "cost per quality-adjusted" or "cost per qaly" or "cost per daly")<br>S2. AB ("cost-effectiveness" or "cost-benefit" or "cost-utility" or "economic evaluation" or "cost per death" or "cost per case" or "cost per infection" or "cost per life" or "cost per disability-adjusted" or "cost per quality-adjusted" or "cost per qaly" or "cost per daly")<br>S3. S1 OR S2<br>S4. TI ("study protocol")<br>S5. S3 NOT S4<br>S6. S5 and PT (Algorithm OR Anecdote OR Bibliography OR Biography OR Book OR Book Chapter OR Book Review OR Brief Item OR Care Plan OR Cartoon OR Case Study OR CEU OR Classification Term OR Code of Ethics OR Commentary OR Computer Program OR Consumer/Patient Teaching Materials OR Critical Path OR Directories OR Doctoral Dissertation OR Editorial OR Evidence-Based Care Sheet OR Exam Questions OR Forms OR Games OR Glossary OR Historical Material OR Interview OR Legal Case OR Letter OR Masters Thesis OR Nurse Practice Acts OR Obituary OR Pamphlet OR Pamphlet Chapter OR Periodical OR Poetry OR Practice Acts OR Proceedings OR Quick Lesson OR Research Instrument Validation OR Response OR Standards OR Statistics OR Teaching Materials OR Tracings OR Trade Publication OR Website)<br>S7. S5 NOT S6

Table S2 Mapping of 25 disease areas onto the Global Burden of Disease (GBD), International Classification of Disease (ICD-10), and search terms used

Health areas developed for this analysis are listed in alphabetical order in the lefthand column. We mapped each component of the Global Burden of Disease (World Health Organization., 2014) onto one health area. The mapping of the ICD-10 codes (World Health Organization., 2011) onto GBD codes is taken from the GBD appendices. Both GBD and ICD-10 definitions were used to inform the development of search terms for each health areas, which were applied as necessary to the titles, abstracts, and/or keywords in the final database of economic evaluations. Underscores ("_") have been used here to show single spaces and question marks ("?") reflect a single wildcard character. GBD: Global Burden of Disease. ICD-10: International Classification of Disease, version 10.

Health area	GBD	ICD-10	Search terms used to identify economic evaluations in this area
**Anaemia**	58: Iron-deficiency anaemia	D50, D64.9	Anaemia, Anemia, Anemic, Anaemic, Iron?deficien, _iron_, iron?supplement,
**Cancer and other neoplasms**	61: Malignant neoplasms, 79: Other neoplasms	C00-C97, D00-D48	Adenocarcinoma, Adenoma, Cancer, carcinoma, chemoradiotherapy, Chemotherapy, Glioblastoma, glioma, Neoplasm, Radiation_therapy, Radiotherapy, Melanoma, Lymphoma, myeloma, neoplastic, Leukaemia, microcalcification, neoplasia, myelodysplas, leukemia, metastatic, sarcoma, paclitaxel, Hematopoietic_Stem_Cell, autologous_stem_cell, cervical_screen, pap_smear, lynch_syndrome, tumour, tumor, breast_reconstruction, metastasis,
**Cardiovascular diseases**	110: Cardiovascular diseases	I00-I99	Angina, Angioplasty, Anticoagulants, aorta, aortic, Arrhythmia, Arrhythmic, arterial, artery, Atrial_Fibrillation, blood_pressure, blood_vessel, cardiac, cardio, carotid, Chest, cholesterol, Coronary, Deep_Vein_Thrombosis, embolism, heart, hypertensi, myocarditis, endocarditis, Myocardial, pulmonary, stroke, aneurysm, circulatory, warfarin, rheumatic, varicose_vein, venous*ulcer, vascular_disease,
**Communicable childhood diseases**	12: Childhood-cluster diseases	A33-A37, B05	chickenpox, Pertussis, Whooping_cough, Diphtheria, Measles, Tetanus, chicken_pox, rubella, immunization,
**Congenital anomalies**	140: Congenital anomalies	Q00-Q99	chromosom, cleft_lip, Dwarfism, cystic_fibrosis, Neural_tube_defect, Cleft_lip, Congenital, cleft_palate, Down_Syndrome, Down's_Syndrome, Down's_Syndrome, disabilities, disabled_children, neural_tube, congenital, Tetralogy_of_Fallot, spina_bifida, trisomy, polydactyl, teratogenic,
**Diabetes**	80: Diabetes mellitus	E10-E14	Diabetes, glucose, diabetic, hyperglycemi,
**Diarrhoeal diseases**	11: Diarrhoeal diseases	A00, A01, A03, A04, A06-A09	diarrhea, diarrhoea, rotavirus, cholera, typhoid, shigell, amoebiasis, amoeba, rotaviral, enteritis, norwalk, adenovir, escherichia_coli, campylobacter, clostridium_difficile, dysentery, giardia, cryptosporid, norovirus,
**Digestive diseases**	121: Digestive diseases	K20-K92	celiac, cholera, coeliac, crohn, Digestive, gallbladder, gallstones, gall?stones, gastric, gastro, helicobacter, ileostomy, colitis, constipation, Appendectomy, appendicitis, hernia_, bowel_, Intestinal_Polyps, Peptic_ulcer, diverticulitis, Cholecystolithiasis, pancreatitis, Cholecystitis, liver_, biliary, duodenal, vomit, ileus, hepatic, inguinal_hernia,

Health area	GBD	ICD-10	Search terms used to identify economic evaluations in this area
**Endocrine, blood, and immune disorders (not diabetes or HIV)**	81: Endocrine, blood, immune disorders	D55-D64 (minus D64.9), D65-D89, E03-E07, E15-E34, E65-E88	graves_disease, Hormones, hyperthyroidism, hypothyroidism, Goiter, Endocrin, Haemophilia, Adrenal, Allerg, Anaphylaxis, hemophilia, thyroid, hematological, neutropenia, Ischaemi, Ischemi, tonsil, thalassaemia, thalassemia, Thrombocytopenia, Fabry_disease, lysosomal, sickle_cell,
**Genitourinary diseases, family planning & fertility**	126: Genitourinary diseases	N00-N64, N75-N76, N80-N98	Gynecolog, Gynaecolog, contraception, contraceptive, embryo, fertility, fertilization, prostat, urologic, urinary, urethral_, genito, Kidney, Urolithiasis, nephrolog, Infertility, infertile, Nephrostomy, dialysis, pyelography, ovulation, urodynamic, ureter, hypogonadism, menstrual, nephropathy, microalbuminur, nephritis, bladder, varicocele,
**HIV/AIDS**	10: HIV/AIDS	B20-B24	Acquired_Immune_Deficiency_Syndrome, CD4, HAART, retroviral, hiv?aids, _hiv_, Human_Immunodeficiency_Virus, cd4_,
**Malaria**	22: Malaria	B50-B54, P37.3, P37.4	bed?net, malaria, bednet, artemesenin,
**Malnutrition (including obesity and exercise)**	54: Nutritional deficiencies (*except 58: Iron-deficiency anaemia*)	E00-E02, E40-E46, E50-E64, D51-D53	bariatric, Body_Mass_Index, Body_Weight, nutrition, Iodine, Vitamin_A, obesity, obese, physical_activity, exercise, pedometer, vegetable, dietary, biofortif, weight_management,
**Maternal and neonatal conditions**	42: Maternal conditions, 49: Neonatal conditions	O00-O99, P00-P96 excl P37.3, P37.4	low?birth?weight, Preterm, Birth, Neonat, Newborn, New-born, Amniocentesis, Birth, caesarean, cesarean, fetal, folic_acid, gestational, preeclampsia, eclampsia, pregnancy, prenatal, abortion, endometrial, obstetric, premature_infant, prematurity, vaginal_deliver,
**Meningitis**	17: Meningitis	A39, G00, G03	mening,
**Mental health, cognition, and developmental and behavioural disorders (including self-harm and addictions)**	82: Mental and behavioural disorders, 161: Self-harm	F04-F99, X41-X42, X45, X60-X84, Y870	ADHD, Agoraphobi, Antidepressant, Antidepressive, Anxiety, autism, Autistic, Schizophreni, Bipolar, _cognition, cognitive, Dementia, depression, substance_use_disorder, opiate_substitution, Eating_Disorder, _Emotions, mental_health, heroin, psychosis, psychotic, Unipolar, cocaine, addiction, Alcohol_use, Drug_use, developmental_disorder, behavioural_disorder, intellectual, behavio?r_disorder, clinically_isolated_syndrome, mentally_ill, Somatoform, depressive_disorder, Alcohol, Drug_Abuse, Drug_Addiction, Narcotic_Control, smoking, substance_abuse, Psychotherapy, mental_illness, Mental_Disorder, suicide, smoker, methadone, methadone, delirium, Nicotine, attention?deficit?hyperactivity?disorder, fear_of, behavior_disorder, cannabis,
**Musculoskeletal diseases (including back and neck pain)**	134: Musculoskeletal diseases	M00-M99	ankle, Bone, Carpal_Tunnel, Cartilage, elbow, fracture, Joint, knee, Ligament, arthritis, Lumbar, Diskectomy, discectomy, musculoskeletal, Physical_Therapy_, osteoporo, fibromyalgia, Spinal, foot, shoulder, orthopedic, hip_replacement, lupus, Gout, low?back_pain,
**Neurological**	94: Neurological	F01-F03, G06 -G98	cerebral, nervous_system, neurological, Epilepsy, Alzheimer, Parkinson,

Health area	GBD	ICD-10	Search terms used to identify economic evaluations in this area
**conditions (including headache and sleep disorders)**	conditions		Epileptic, Multiple_sclerosis, Migraine, headache, sleep, Myasthenia_gravis, thymectomy, neurosurgery, neuropath, chronic_fatigue_syndrome, neuralgia,
**Other infectious diseases (including encephalitis, hep A, B, C, and other parasitic and vector-borne diseases, and nematode infections)**	18: Encephalitis, 19: Acute hepatitis B, 20: Acute hepatitis C, 21: Parasitic and vector diseases (*except 22: Malaria*), 33: Intestinal nematode infections, 37: Other infectious diseases	A83-A86, B94.1, G04, B16-B19 (minus B17.1, B18.2), B17.1, B18.2, A30, A71, A82, A90-A91, B55-B57, B65, B73, B74.0-B74.2, B76-B77, B79, A02, A05, A20-A28, A31, A32, A38, A40-A49, A65-A70, A74-A79, A80-A81, A87-A89, A92-A99, B00-B04, B06-B15, B25-B49, B58-B60, B64, B66-B72, B74.3-B74.9, B75,B78, B80-B89, B91-B99 (minus B94.1)	Encephaliti, Dengue, lyme_, deworming, hepatitis, hep_b, hep_c, Trypanosomiasis, Chagas, Schistosom, Leishmania, Lymphatic_filariasis, Onchocerciasis, Leprosy, leprous, Trachoma, Rabies, Ascariasis, Trichuriasis, Hookworm, hep_a, rubella, herpes_zoster, clostridium, Staphylococc, Bacteremia, hospital-acquired_infection, septic_shock, sepsis, Staphylococc, scabies, systemic_Candida_infection, cytomegalovirus, infection_control, hcv, Creutzfeldt?Jakob, _invasive_Candid, Helminth, roundworm, antimicrobial,
**Respiratory diseases**	38: Respiratory infections, 117: Respiratory diseases	J00-J22, H65-H68,P23, U04, J30-J98	Respiratory, Pulmonary, Lung, Bronchial, Trachea, Bronchitis, Airway, Asthma, H1N1, Influenza, bird_flu, avian_flu, interstit, Pleural_effusion, sore_throat, pneumonia, Respiration, pneumococcal, Haemophilus, breathing, pharyngitis, pneumonia,
**Sense organ diseases**	102: Sense organ diseases	H00-H61, H69-H93	Blindness, Cataract, cochlear, deafness, eye_, Glaucoma, hearing, Macular, rhino, Nasolacrimal, Refractive_error, vision_loss, hearing_loss, canaloplasty, Trabeculectomy, retina, Ophthalmolog, keratoplasty, otitis_media,
**Skin and oral conditions**	133: Skin diseases, 147: Oral conditions	L00-L98, K00-K14	debridement, Dental, Dentistry, Denture, gingival, Edentulism, Periodontal, peritonsillar, Dentition, Orthodontics, Dermatitis, Dermatolog, Eczema, skin_disease, tinea, psoriasis, dermatophytic, plantar_wart, skin?graft, soft_tissue, foam_dressing, pressure_ulcer,
**Sexually Transmitted Infections (not HIV)**	4: STDs excluding HIV	A50-A64, N70-N73	chlamydia, condoms, Gonorrhea, Papillomavirus, Syphilis, Gonorrhoea, Trichomoniasis, Sexually_Transmitted, hpv,
**Tuberculosis**	3: Tuberculosis	A15-A19, B90	antitubercular, BCG, TB, tuberculos, bacille_Calmette-Guerin,
**Wounds and injuries**	151: Injuries (*except 161: Self-harm*)	V01-X59, Y871-Y89,	Injury, Injuries, Accident, Burn, violence, Poisoning, Drown, child_abuse, domestic_abuse, Domestic_Violence, trauma, fall_prevention, falls_prevention, venom, antidote, whiplash, _radon_, road_safety,

Table S3 Classification of journal types

The following is a comprehensive list of how we classified journals publishing at least one economic evaluation meeting our criteria.

Health economics, policy, services, and/or social science journals	
Administration and Policy in Mental Health and Mental Health Services Research	International Journal of Technology Assessment in Health Care
AIDS and behavior	Israel Journal of Health Policy Research
Alter	Journal d'Economie Medicale
American Health and Drug Benefits	Journal of Benefit-Cost Analysis
Applied Health Economics and Health Policy	Journal of health economics
Behaviour research and therapy	JOURNAL OF HEALTH SERVICES RESEARCH and POLICY
BMC HEALTH SERVICES RESEARCH	Journal of Medical Economics
BMC Medical Informatics and Decision Making	Journal of Mental Health Policy and Economics
British Journal of Health Care Management	Journal of Nursing Management
Bulletin of the World Health Organization	JOURNAL OF NUTRITION EDUCATION AND BEHAVIOR
Cancer Management and Research	Journal of Pain and Symptom Management
CHILDREN AND YOUTH SERVICES REVIEW	Journal of Pharmaceutical Health Services Research
ClinicoEconomics and Outcomes Research	Journal of Public Health Management and Practice
Cost Effectiveness and Resource Allocation	Medical Decision Making
Decision Sciences	Mediterranean Journal of Social Sciences
Epilepsy and Behavior	Ontario Health Technology Assessment Series
European Journal of Health Economics	Open Pharmacoeconomics and Health Economics Journal
European Review of Agricultural Economics	PharmacoEconomics
Expert review of pharmacoeconomics and outcomes research	PharmacoEconomics - Italian Research Articles
Gesundheitsokonomie und Qualitatsmanagement	Pharmacoeconomics - Spanish Research Articles
GMS health technology assessment	Population Health Management
Health Affairs	Psychological Services
Health Economics	Research in Social and Administrative Pharmacy
Health Economics Review	Revista medica del Instituto Mexicano del Seguro Social
Health Policy	Social Psychiatry and Psychiatric Epidemiology
Health Policy and Planning	South African Journal of Economic and Management Sciences
Health Policy and Technology	Substance abuse treatment, prevention, and policy
Health Services Research	Therapeutics and Clinical Risk Management
Health Technology Assessment	Value in Health
Healthcare Policy	Value in Health Regional Issues
International Journal of Behavioral Nutrition and Physical Activity	Vascular Health and Risk Management
International Journal of Drug Policy	
**Other**	
American Water Works Association Journal	Journal of Water and Health
Child Abuse and Neglect	MATHEMATICAL MODELLING OF NATURAL PHENOMENA
Disasters	PLoS One
Environment International	Traffic Injury Prevention
European Journal of Operational Research	
Journal of interpersonal violence	
**Biomedical journals**	
[Rinsho ketsueki] The Japanese journal of clinical hematology	Iowa Orthopaedic Journal
Academic Emergency Medicine	Iranian journal of neurology
Academic Pediatrics	Iranian Journal of Pediatrics
Acta Chirurgiae Orthopaedicae et Traumatologiae Cechoslovaca	Iranian Journal of Pharmaceutical Research
Acta Chirurgica Belgica	Iranian Journal of Radiology
	Iranian Red Crescent Medical Journal
	Irish Journal of Medical Science

Acta clinica Belgica	ISRN Gastroenterology
Acta gastroenterologica Latinoamericana	ISRN Obstetrics and Gynecology
Acta Medica Indonesiana	Italian Journal of Public Health
Acta neurochirurgica	JACC: Heart Failure
Acta Neurologica Scandinavica	JAMA
Acta Neuropsychiatrica	JAMA Ophthalmology
Acta Obstetricia et Gynecologica Scandinavica	JAMA Pediatrics
Acta Oncologica	Japanese Journal of Ophthalmology
Acta Ophthalmologica	Japanese Pharmacology and Therapeutics
Acta Oto-Laryngologica	Joint Commission Journal on Quality and Patient Safety
Acta Psychiatrica Scandinavica	Joint, Bone, Spine
Acta Radiologica	Jornal Portugues de Gastrenterologia
Actas dermo-sifiliograficas	Journal de Mycologie Medicale
Actas Urologicas Espanolas	Journal for Healthcare Quality
Acupuncture in Medicine	Journal of Acquired Immune Deficiency Syndromes
Addiction	Journal of Adolescent Health
Advances in Clinical and Experimental Medicine	Journal of Advanced Nursing
Advances in Skin and Wound Care	Journal of affective disorders
Advances in Therapy	Journal of Aging Research
Aesthetic Surgery Journal	Journal of Allergy and Clinical Immunology
African health sciences	Journal of Alternative and Complementary Medicine
African Journal of AIDS Research	Journal of Antivirals and Antiretrovirals
African Journal of Urology	Journal of Anxiety Disorders
Age and Ageing	Journal of Arthroplasty
AIDS	Journal of Arthropod-Borne Diseases
AIDS Care	Journal of Asthma
Alcohol and Alcoholism	Journal of bone and joint surgery
Alcoholism Clinical and Experimental Research	Journal of Bone and Mineral Research
Alimentary Pharmacology and Therapeutics	Journal of Brain Science
Allergologie	Journal of Bronchology and Interventional Pulmonology
Alzheimer's and Dementia	
American heart journal	Journal of Burn Care and Research
American Journal of Cardiology	Journal of Cancer
American Journal of Cardiovascular Drugs	Journal of Cancer Epidemiology
American Journal of Clinical Dermatology	Journal of Cardiothoracic Surgery
American Journal of Clinical Oncology	Journal of Cardiovascular Computed Tomography
American Journal of Emergency Medicine	Journal of Cardiovascular Electrophysiology
American Journal of Gastroenterology	Journal of Cardiovascular Magnetic Resonance
American Journal of Geriatric Psychiatry	Journal of Cardiovascular Medicine
American Journal of Health-System Pharmacy	Journal of Cardiovascular Nursing
AMERICAN JOURNAL OF HYPERTENSION	Journal of Cataract and Refractive Surgery
American Journal of Industrial Medicine	Journal of Child and Adolescent Substance Abuse
American Journal of Infection Control	Journal of Child and Family Studies
American Journal of Kidney Diseases	Journal of Children's Orthopaedics
American Journal of Managed Care	Journal of Clinical Apheresis
American Journal of Medical Genetics Part A	Journal of Clinical Endocrinology and Metabolism
American Journal of Medicine	Journal of clinical gastroenterology
American Journal of Neuroradiology	Journal of Clinical Hypertension
American Journal of Obstetrics and Gynecology	Journal of clinical lipidology
American journal of ophthalmology	JOURNAL OF CLINICAL MICROBIOLOGY
American Journal of Perinatology	Journal of clinical nursing
American Journal of Pharmacy Benefits	Journal of Clinical Oncology
American Journal of Physical Medicine and Rehabilitation	Journal of Clinical Periodontology
	Journal of Clinical Psychiatry
American Journal of Preventive Medicine	Journal of Clinical Sleep Medicine
American Journal of Public Health	Journal of Clinical Ultrasound
American Journal of Rhinology and Allergy	Journal of Clinical Virology

American Journal of Roentgenology	Journal of Cognitive and Behavioral Psychotherapies
American Journal of Speech-Language Pathology	Journal of community health
American journal of sports medicine	Journal of Comparative Effectiveness Research
American Journal of Surgery	JOURNAL OF CROHNS and COLITIS
AMERICAN JOURNAL OF THERAPEUTICS	Journal of Crohn's and Colitis
American Journal of Transplantation	Journal of Cystic Fibrosis
American Journal of Tropical Medicine and Hygiene	Journal of Dental Research
Anesthesiology	Journal of Dermatological Treatment
Angiology	Journal of Endourology
Annali di igiene : medicina preventiva e di comunita	Journal of Endovascular Therapy
Annals of Allergy, Asthma and Immunology	JOURNAL OF EPIDEMIOLOGY AND COMMUNITY HEALTH
Annals of cardiothoracic surgery	
Annals of Emergency Medicine	Journal of evaluation in clinical practice
Annals of General Psychiatry	Journal of Food and Drug Analysis
Annals of Hematology	Journal of Food Protection
Annals of Internal Medicine	JOURNAL OF FOOT AND ANKLE RESEARCH
Annals of Nuclear Medicine	JOURNAL OF GASTROENTEROLOGY AND HEPATOLOGY
Annals of Oncology	Journal of Gastrointestinal Cancer
Annals of Pharmacotherapy	Journal of Gastrointestinal Surgery
Annals of Plastic Surgery	Journal of General Internal Medicine
Annals of rehabilitation medicine	Journal of Global Health
Annals of Surgery	Journal of gynecologic oncology
Annals of Surgical Oncology	Journal of hand surgery
Annals of the Rheumatic Diseases	journal of headache and pain
Annals of the Royal College of Surgeons of England	Journal of Hearing Science
Annals of Thoracic Surgery	Journal of Heart and Lung Transplantation
Annals of Vascular Surgery	JOURNAL OF HEPATOLOGY
Antimicrobial Agents and Chemotherapy	Journal of Hospital Infection
Antiviral therapy	Journal of Hospital Medicine
ANZ Journal of Surgery	Journal of Hypertension
Archives of Disease in Childhood	Journal of Infection
Archives of Gynecology and Obstetrics	Journal of Infectious Diseases
Archives of Internal Medicine	Journal of Interventional Cardiology
Archives of Iranian Medicine	Journal of Korean Academy of Nursing Administration
Archives of Medical Research	Journal of Korean Medical Science
Archives of Medical Science	Journal of long-term effects of medical implants
Archives of Ophthalmology	Journal of Lower Genital Tract Disease
Archives of Pathology and Laboratory Medicine	Journal of Managed Care Medicine
Archives of Pediatrics and Adolescent Medicine	Journal of Managed Care Pharmacy
Archives of Surgery	journal of maternal-fetal and neonatal medicine
ARCHIVOS DE BRONCONEUMOLOGIA	Journal of Medical Colleges of PLA
Archivos de Neurociencias	Journal of Medical Internet Research
Arquivos brasileiros de cardiologia	Journal of Mental Health
Arquivos Brasileiros de Oftalmologia	Journal of microbiology, immunology, and infection = Wei mian yu gan ran za zhi
Arquivos de Gastroenterologia	
Arthritis Care and Research	Journal of Nervous and Mental Disease
Arthroscopy	Journal of Neurology
ARYA Atherosclerosis	Journal of Neurology Neurosurgery and Psychiatry
Asian Biomedicine	Journal of Neuro-Ophthalmology
Asian Pacific Journal of Cancer Prevention	Journal of neurosurgery
Asian Pacific Journal of Tropical Disease	Journal of neurosurgery. Spine
Asia-Pacific Journal of Clinical Oncology	Journal of Neurosurgery: Spine
Asia-Pacific Journal of Public Health	JOURNAL OF NEUROSURGERY-SPINE
Atencion Farmaceutica	Journal of Nuclear Medicine
Atencion Primaria	Journal of Nursing Scholarship
Australian and New Zealand Journal of Obstetrics and Gynaecology	Journal of Nutrition
	JOURNAL OF OBSTETRICS AND GYNAECOLOGY

Australian and New Zealand journal of public health	Journal of Obstetrics and Gynaecology Canada
Australian Health Review	Journal of Occupational and Environmental Medicine
Australian Journal of Primary Health	Journal of Occupational Rehabilitation
Autism	Journal of Oncology Pharmacy Practice
Biochemia medica	Journal of Oncology Practice
BioDrugs	Journal of Orthopaedic Research
Biologics in Therapy	Journal of orthopaedic trauma
Biology of Blood and Marrow Transplantation	Journal of Otolaryngology - Head and Neck Surgery
BioMed research international	Journal of Pain and Palliative Care Pharmacotherapy
Biomedica	Journal of pediatric ophthalmology and strabismus
BIOMEDICAL ENGINEERING-BIOMEDIZINISCHE TECHNIK	Journal of Pediatrics
	Journal of Perinatology
Biomedical Journal	Journal of Periodontology
Biosecurity and Bioterrorism	Journal of Pharmacy Practice
BIOSYSTEMS	Journal of Plastic, Reconstructive and Aesthetic Surgery
BIOTECHNOLOGY and BIOTECHNOLOGICAL EQUIPMENT	Journal of Population Therapeutics and Clinical Pharmacology
BJOG: An International Journal of Obstetrics and Gynaecology	Journal of Practical Oncology
	Journal of primary care and community health
BJU International	Journal of Psychiatric Research
Blood purification	Journal of Psychosomatic Research
BMC Anesthesiology	Journal of Public Health
BMC Cancer	JOURNAL OF PUBLIC HEALTH DENTISTRY
BMC Cardiovascular Disorders	Journal of Radiation Research
BMC Clinical Pharmacology	Journal of Rehabilitation Medicine
BMC Complementary and Alternative Medicine	Journal of research in health sciences
BMC family practice	Journal of Sexual Medicine
BMC Gastroenterology	Journal of Shoulder and Elbow Surgery
BMC infectious diseases	Journal of Spinal Disorders and Techniques
BMC Medical Research Methodology	Journal of Stroke and Cerebrovascular Diseases
BMC Medicine	Journal of Studies on Alcohol and Drugs
BMC Musculoskeletal Disorders	Journal of Substance Abuse Treatment
BMC Neurology	Journal of Surgical Oncology
BMC ophthalmology	Journal of Surgical Research
BMC Pediatrics	Journal of Telemedicine and Telecare
BMC pregnancy and childbirth	Journal of the Academy of Nutrition and Dietetics
BMC Psychiatry	Journal of the American Academy of Audiology
BMC PUBLIC HEALTH	Journal of the American Academy of Dermatology
BMC research notes	Journal of the American College of Cardiology
BMJ	Journal of the American College of Surgeons
BMJ Open	JOURNAL OF THE AMERICAN GERIATRICS SOCIETY
BMJ quality and safety	Journal of the American Medical Directors Association
BMJ supportive and palliative care	Journal of the American Medical Informatics Association
Boletin Medico del Hospital Infantil de Mexico	
Bone	Journal of the American Pharmacists Association
bone and joint journal	Journal of the American Society of Nephrology
Brachytherapy	Journal of the Balkan Union of Oncology
Brazilian Journal of Infectious Diseases	Journal of the European Academy of Dermatology and Venereology
Brazilian Journal of Pharmaceutical Sciences	
Breast Cancer Research and Treatment	JOURNAL OF THE FORMOSAN MEDICAL ASSOCIATION
Breast Cancer: Targets and Therapy	Journal of the International Association of Providers of AIDS Care
Breast Care	
Breastfeeding Medicine	Journal of the Medical Association of Thailand
British Journal of Anaesthesia	Journal of the National Cancer Institute
British Journal of Cancer	Journal of the National Comprehensive Cancer Network
British Journal of Dermatology	
British Journal of General Practice	Journal of the Neurological Sciences

British Journal of Haematology<br>British Journal of Ophthalmology<br>British Journal of Psychiatry<br>British Journal of Sports Medicine<br>British Journal of Surgery<br>Bulletin du Cancer<br>Cadernos de Saude Publica<br>CADTH technology overviews<br>Canadian Journal of Cardiology<br>Canadian Journal of Infectious Diseases and Medical Microbiology<br>Canadian Journal of Ophthalmology<br>Canadian Journal of Surgery<br>Canadian Journal of Urology<br>Canadian Journal on Aging<br>Canadian Medical Association Journal<br>Canadian Urological Association Journal<br>Cancer<br>Cancer Causes and Control<br>Cancer Epidemiology Biomarkers and Prevention<br>Cancer Epidemiology, Biomarkers and Prevention<br>Cancer Prevention Research<br>Cardiogenetics<br>CardioVascular and Interventional Radiology<br>Cardiovascular Drugs and Therapy<br>Cardiovascular journal of Africa<br>Cardiovascular Therapeutics<br>Caries Research<br>Catheterization and Cardiovascular Interventions<br>Cerebrovascular Diseases<br>Ceska Gynekologie<br>Chest<br>Child and Adolescent Psychiatry and Mental Health<br>Childhood Obesity<br>Chinese Journal of Cancer Prevention and Treatment<br>Chinese Journal of Clinical Nutrition<br>Chinese Journal of Clinical Oncology<br>Chinese Journal of Evidence-Based Medicine<br>Chinese Journal of Lung Cancer<br>Chinese Journal of New Drugs<br>Chinese Journal of Oncology<br>Chinese Journal of Schistosomiasis Control<br>Chinese Journal of Tissue Engineering Research<br>Chinese Pharmaceutical Journal<br>Chinese Preventive Medicine<br>Chongqing Medicine<br>Ciencia and saude coletiva<br>Ciencia y Enfermeria<br>Circulation<br>Circulation: Cardiovascular Quality and Outcomes<br>Circulation: Heart Failure<br>CIRCULATION-CARDIOVASCULAR QUALITY AND OUTCOMES<br>Cirugia Espanola<br>Cirugia y Cirujanos<br>Clinica e Investigacion en Ginecologia y Obstetricia<br>Clinical and Experimental Nephrology	Journal of the Pakistan Medical Association<br>Journal of the Royal Society Interface<br>Journal of the Royal Society of Medicine<br>Journal of Theoretical Biology<br>Journal of Thoracic and Cardiovascular Surgery<br>Journal of Thoracic Oncology<br>Journal of Thrombosis and Haemostasis<br>Journal of thrombosis and thrombolysis<br>Journal of Traditional Chinese Medicine<br>Journal of Trauma and Acute Care Surgery<br>Journal of Urban Health<br>Journal of Urology<br>Journal of vascular and interventional neurology<br>Journal of Vascular and Interventional Radiology<br>Journal of Vascular Nursing<br>Journal of Vascular Surgery<br>JOURNAL OF VIRAL HEPATITIS<br>Journal of Women's Health<br>Journal of wound care<br>Kardiologia Polska<br>KARDIOLOGIYA<br>Kidney and Blood Pressure Research<br>Klimik Dergisi<br>Klinische Monatsblatter fur Augenheilkunde<br>Klinische P+ndiatrie<br>Knee Surgery, Sports Traumatology, Arthroscopy<br>Korean Journal of Thoracic and Cardiovascular Surgery<br>La Radiologia medica<br>Lancet<br>Lancet Global Health<br>Lancet Infectious Diseases<br>Laryngoscope<br>Leukemia and Lymphoma<br>Lin chuang er bi yan hou tou jing wai ke za zhi = Journal of clinical otorhinolaryngology, head, and neck surgery<br>Liver Transplantation<br>Lung Cancer<br>Malaria Journal<br>Managed Care<br>Maternal and Child Health Care of China<br>Mathematical Biosciences and Engineering<br>Medical Care<br>MEDICAL HYPOTHESES<br>MEDICAL JOURNAL OF AUSTRALIA<br>Medical Journal of Chinese People's Liberation Army<br>Medical Journal of Malaysia<br>Medicina Preventiva<br>Medicine, Health Care and Philosophy<br>Methodist DeBakey cardiovascular journal<br>Midwifery<br>Modern Preventive Medicine<br>Molecular and Clinical Oncology<br>Molecular Diagnosis and Therapy<br>MOVEMENT DISORDERS<br>Multiple Sclerosis<br>Mycoses

Clinical and experimental obstetrics and gynecology<br>Clinical and Experimental Rheumatology<br>Clinical and translational allergy<br>Clinical Breast Cancer<br>Clinical Cardiology<br>Clinical drug investigation<br>Clinical Gastroenterology and Hepatology<br>Clinical Infectious Diseases<br>Clinical Journal of Pain<br>Clinical Journal of the American Society of Nephrology<br>Clinical Laboratory<br>Clinical Medicine Insights: Therapeutics<br>Clinical Microbiology and Infection<br>Clinical Nephrology<br>Clinical Neurology and Neurosurgery<br>Clinical Neurophysiology<br>Clinical Nutrition<br>Clinical Oncology<br>Clinical orthopaedics and related research<br>Clinical Otolaryngology<br>Clinical pediatrics<br>Clinical Pharmacology and Therapeutics<br>Clinical Rehabilitation<br>Clinical Research in Cardiology<br>CLINICAL RHEUMATOLOGY<br>Clinical Therapeutics<br>Clinical Transplantation<br>Clinical Trials<br>Clinics<br>CNS Drugs<br>Cochrane Database of Systematic Reviews<br>Cocuk Enfeksiyon Dergisi<br>Colorectal Disease<br>Community Dental Health<br>Community Dentistry and Oral Epidemiology<br>Community Oncology<br>Contact Dermatitis<br>Contraception<br>Crisis<br>Critical Care Medicine<br>Critical Pathways in Cardiology<br>Current Alzheimer Research<br>Current Medical Research and Opinion<br>Current Oncology<br>Danish Medical Journal<br>DARU<br>Das Gesundheitswesen<br>Dementia and Geriatric Cognitive Disorders<br>Dermatology<br>Dermatology and therapy<br>Diabetes and Vascular Disease Research<br>Diabetes care<br>Diabetes Research and Clinical Practice<br>Diabetes Technology and Therapeutics<br>Diabetes Therapy<br>Diabetes, Obesity and Metabolism<br>Diabetic Medicine	Nan fang yi ke da xue xue bao = Journal of Southern Medical University<br>National Medical Journal of China<br>Nephrology Dialysis Transplantation<br>Netherlands Journal of Medicine<br>Neurologia<br>Neurologia medico-chirurgica<br>Neurologist<br>Neurology<br>Neuro-oncology<br>Neurosurgery<br>Neurourology and Urodynamics<br>New Biotechnology<br>NEW ENGLAND JOURNAL OF MEDICINE<br>Nicotine and Tobacco Research<br>North Carolina medical journal<br>Nutrition and Diabetes<br>OBESITY<br>Obesity Research and Clinical Practice<br>Obesity surgery<br>Obstetrics and Gynecology<br>Occupation and Health<br>Occupational Medicine<br>Ochsner journal<br>Oncologist<br>Oncology<br>Open Respiratory Medicine Journal<br>Open Rheumatology Journal<br>Ophthalmic Epidemiology<br>Ophthalmologica<br>Ophthalmology<br>Oral Oncology<br>Orphanet journal of rare diseases<br>Orthopedics<br>Osteoarthritis and Cartilage<br>Osteoporosis International<br>Otolaryngology - Head and Neck Surgery<br>Pacing and Clinical Electrophysiology<br>Paediatric Anaesthesia<br>Paediatrics and Child Health<br>PAEDIATRICS AND INTERNATIONAL CHILD HEALTH<br>Pain Medicine<br>Pain physician<br>Pain Practice<br>Pan African Medical Journal<br>PARASITES and VECTORS<br>Payesh Health Monitor<br>PEDIATRIC ALLERGY AND IMMUNOLOGY<br>Pediatric Cardiology<br>Pediatric Drugs<br>PEDIATRIC EMERGENCY CARE<br>Pediatric Infectious Disease Journal<br>Pediatric obesity<br>Pediatric Transplantation<br>Pediatrics<br>Pediatrics International<br>Perioperative Medicine

Digestive and Liver Disease<br>Digestive diseases and sciences<br>Digestive Surgery<br>Diseases of the Colon and Rectum<br>DMW Deutsche Medizinische Wochenschrift<br>Drug and Alcohol Dependence<br>Drug Metabolism and Pharmacokinetics<br>Drugs and Aging<br>Ear and hearing<br>ecancermedicalscience<br>Eksperimental'naia i klinicheskaia gastroenterologiia = Experimental and clinical gastroenterology<br>EMERGENCIAS<br>Emergency Medicine Journal<br>Emerging Infectious Diseases<br>Endoscopy<br>Enfermedades Infecciosas y Microbiologia Clinica<br>Epidemiology and Infection<br>Epidemiology and Psychiatric Science<br>Epilepsia<br>Epilepsy Research<br>Europace<br>European Annals of Otorhinolaryngology, Head and Neck Diseases<br>European Child and Adolescent Psychiatry<br>European Heart Journal<br>EUROPEAN HEART JOURNAL-CARDIOVASCULAR IMAGING<br>European Journal of Cancer<br>European Journal of Cardio-Thoracic Surgery<br>European Journal of Clinical Microbiology and Infectious Diseases<br>European Journal of Clinical Nutrition<br>European Journal of Gastroenterology and Hepatology<br>European Journal of Haematology<br>European Journal of Heart Failure<br>European Journal of Hospital Pharmacy: Science and Practice<br>European Journal of Human Genetics<br>European Journal of Integrative Medicine<br>European Journal of Neurology<br>European Journal of Nuclear Medicine and Molecular Imaging<br>EUROPEAN JOURNAL OF OBSTETRICS and GYNECOLOGY AND REPRODUCTIVE BIOLOGY<br>European Journal of Obstetrics Gynecology and Reproductive Biology<br>European Journal of Orthopaedic Surgery and Traumatology<br>European Journal of Paediatric Neurology<br>EUROPEAN JOURNAL OF PAIN<br>European Journal of Preventive Cardiology<br>European journal of public health<br>European Journal of Radiology<br>European Journal of Vascular and Endovascular Surgery<br>European Neurology<br>European Neuropsychopharmacology	Peritoneal Dialysis International<br>Personalized Medicine<br>Pharmacogenetics and Genomics<br>Pharmacogenomics<br>Pharmacotherapy<br>Pharmazie<br>Physis: Revista de Saude Coletiva<br>Plastic and reconstructive surgery<br>PLOS MEDICINE<br>PloS Neglected Tropical Diseases<br>PM and R<br>Polski Merkuriusz Lekarski<br>Postepy Dermatologii I Alergologii<br>Postgraduate medicine<br>Practical Pharmacy and Clinical Remedies<br>Prenatal Diagnosis<br>Presse Medicale<br>Preventing chronic disease<br>PREVENTION SCIENCE<br>Preventive Medicine<br>Primary care diabetes<br>Primary Care Respiratory Journal<br>Proceedings of the National Academy of Sciences of the United States of America<br>Progresos de Obstetricia y Ginecologia<br>Progress in Modern Biomedicine<br>Progress in Neuro-Psychopharmacology and Biological Psychiatry<br>Prostate cancer and prostatic diseases<br>Psychiatrische Praxis<br>Psychological Medicine<br>Psychologische Rundschau<br>Psycho-Oncology<br>Psychosomatics<br>Psychotherapy Research<br>Public health nutrition<br>QJM<br>QUALITY OF LIFE RESEARCH<br>Radiol. bras<br>Radiological Physics and Technology<br>Radiology<br>Rational Pharmacotherapy in Cardiology<br>Rehabilitacion<br>Renal Failure<br>Reproductive biomedicine online<br>Research in Autism Spectrum Disorders<br>Research Journal of Pharmacy and Technology<br>Respiratory medicine<br>Reumatologia<br>Reumatologia Clinica<br>Revista Brasileira de Cardiologia Invasiva<br>Revista Brasileira de Cirurgia Cardiovascular<br>Revista Clinica de Medicina de Familia<br>Revista clinica espanola<br>Revista Colombiana de Cardiologia<br>Revista Colombiana de Obstetricia y Ginecologia<br>REVISTA DA ASSOCIACAO MEDICA BRASILEIRA

European Radiology<br>European Respiratory Journal<br>European Review for Medical and Pharmacological Sciences<br>European Spine Journal<br>European Urology<br>Evaluation and Program Planning<br>Evidence Based Medicine<br>Experimental and Therapeutic Medicine<br>Expert Review of Anticancer Therapy<br>Familial Cancer<br>Farmacia Hospitalaria<br>Female pelvic medicine and reconstructive surgery<br>Fertility and Sterility<br>Fisioterapia<br>Food and Nutrition Bulletin<br>Foot and Ankle International<br>Foot and Ankle Surgery<br>Fortschritte auf dem Gebiete der Rontgenstrahlen und der Nuklearmedizin<br>Forum of Clinical Oncology<br>Frontiers in oncology<br>Gastroenterologia y Hepatologia<br>Gastroenterology<br>Gastrointestinal Endoscopy<br>Gazzetta Medica Italiana<br>Genetics in Medicine<br>Gerodontology<br>Ginecologia y Obstetricia de Mexico<br>GLOBAL HEALTH ACTION<br>Global Journal of Health Science<br>Global Public Health<br>Gut<br>Gut and Liver<br>Gynecologic Endocrinology<br>Gynecologic Oncology<br>Haematologica<br>Haemophilia<br>Hawaii Journal of Medicine and Public Health<br>Health<br>Health Outcomes Research in Medicine<br>Health promotion international<br>Health Psychology<br>Heart<br>Heart Lung and Circulation<br>Heart Rhythm<br>Hellenic Journal of Cardiology<br>Hematological Oncology<br>Hematology/ Oncology and Stem Cell Therapy<br>Hepatitis Monthly<br>Hepato-Gastroenterology<br>HEPATOLOGY<br>Hepatology Research<br>Hinyokika kiyo. Acta urologica Japonica<br>HIP International<br>HIV Clinical Trials<br>HIV MEDICINE	Revista de Associacao Medica Brasileira<br>Revista de enfermeria (Barcelona, Spain)<br>Revista de la Sociedad Espanola del Dolor<br>Revista de Salud Publica<br>Revista de Saude Publica<br>Revista espanola de anestesiologia y reanimacion<br>Revista Espanola de Cardiologia<br>Revista Espanola de Cirugia Ortopedica y Traumatologia<br>Revista Espanola de Quimioterapia<br>Revista Espanola de Salud Publica<br>Revista Mexicana de Neurociencia<br>Revista Panamericana de Salud Publica<br>Revista Portuguesa de Cardiologia<br>Revista Salud Publica (Bogota)<br>Revue de Medecine Interne<br>Revue de Neuropsychologie, Neurosciences Cognitives et Cliniques<br>Revue des maladies respiratoires<br>Revue du Rhumatisme (Edition Francaise)<br>Rheumatology<br>Rheumatology International<br>Risk Analysis<br>RUSSIAN JOURNAL OF CARDIOLOGY<br>Salud Publica de Mexico<br>Salud(i)Ciencia<br>Sarcoma<br>Saudi Medical Journal<br>Scandinavian Cardiovascular Journal<br>SCANDINAVIAN JOURNAL OF INFECTIOUS DISEASES<br>SCANDINAVIAN JOURNAL OF PUBLIC HEALTH<br>Scandinavian Journal of Urology and Nephrology<br>Scandinavian Journal of Work Environment and Health<br>Schizophrenia Research<br>Semergen<br>Seminars in Spine Surgery<br>Sex Education<br>Sexual Health<br>Sexually transmitted diseases<br>Sexually Transmitted Infections<br>Singapore Medical Journal<br>Sleep<br>South African Journal of Obstetrics and Gynaecology<br>South African Medical Journal<br>Spine<br>Spine Deformity<br>Spine Journal<br>SpringerPlus<br>STOCHASTIC ENVIRONMENTAL RESEARCH AND RISK ASSESSMENT<br>Stroke<br>Supportive Care in Cancer<br>Surgery<br>Surgical Endoscopy<br>Surgical Endoscopy and Other Interventional Techniques<br>Surgical Laparoscopy, Endoscopy and Percutaneous

Hong Kong Medical Journal
Hormone Research in Paediatrics
Hospital Practice
HPB
Human Reproduction
Human Vaccines
Human Vaccines and Immunotherapeutics
Imaging in Medicine
Indian Journal of Community Medicine
Indian Journal of Dermatology
Indian Journal of Medical and Paediatric Oncology
Indian Journal of Pharmacology
Indian journal of public health
Infant, Child and Adolescent Nutrition
Infection
Infection Control and Hospital Epidemiology
Infectious Diseases in Obstetrics and Gynecology
Inflammatory Bowel Diseases
Influenza and other Respiratory Viruses
Injury
Injury Prevention
Insights into Imaging
Intensive care medicine
Internal medicine journal
International Brazilian Journal of Urology
International Forum of Allergy and Rhinology
International Health
International Journal for Quality in Health Care
International journal of Alzheimer's disease
International Journal of Antimicrobial Agents
International Journal of Cancer
International Journal of Cardiology
International journal of chronic obstructive pulmonary disease
International journal of clinical pharmacy
International journal of clinical practice
International Journal of COPD
International Journal of Dermatology
International Journal of Drug Development and Research
International Journal of Eating Disorders
International Journal of Environmental Research and Public Health
International Journal of Geriatric Psychiatry
International Journal of Group Psychotherapy
International Journal of Gynecological Cancer
International Journal of Gynecology and Obstetrics
International Journal of Health Care Quality Assurance
International journal of inflammation
International Journal of Medical Engineering and Informatics
International Journal of Nursing Studies
International Journal of Obesity
International journal of pediatric otorhinolaryngology
International Journal of Pharmaceutical Sciences Review and Research
International Journal of Pharmacology

Techniques
Swiss Medical Weekly
Technology in Cancer Research and Treatment
Telemedicine and e-Health
Theoretical biology and medical modelling
Therapeutic Advances in Psychopharmacology
Thorax
Thrombosis and Haemostasis
Thrombosis Journal
Thrombosis Research
Tijdschrift voor Geneeskunde
Tobacco Control
Toxicon
Toxins
TRANSACTIONS OF THE ROYAL SOCIETY OF TROPICAL MEDICINE AND HYGIENE
Transfusion
Transfusion and Apheresis Science
TRANSPLANT INTERNATIONAL
Transplantation
Transplantation Proceedings
Trials
Tropical Medicine and International Health
Tumor
Turkderm Deri Hastaliklari ve Frengi Arsivi
TURKISH JOURNAL OF MEDICAL SCIENCES
Ultrasound in Obstetrics and Gynecology
University of Toronto Medical Journal
Vaccine
Vakcinologie
Vascular and endovascular surgery
Vector-Borne and Zoonotic Diseases
Vestnik Dermatologii i Venerologii
Vojnosanitetski pregled. Military-medical and pharmaceutical review
Voprosy Onkologii
Vox sanguinis
Wiener Klinische Wochenschrift
Wiener Medizinische Wochenschrift
Work
World Chinese Journal of Digestology
World Journal of Emergency Surgery
World Journal of Gastroenterology
World journal of surgery
World Journal of Surgical Oncology
World Neurosurgery
Wounds
ZDRAVSTVENO VARSTVO
Zeitschrift fur Evidenz Fortbildung und Qualitat im Gesundheitswesen
Zeitschrift fur Gerontologie und Geriatrie
Zhongguo Shiyong Neike Zazhi / Chinese Journal of Practical Internal Medicine
Zhongguo Xinyao yu Linchuang Zazhi
Zhongguo Zhong xi yi jie he za zhi Zhongguo Zhongxiyi jiehe zazhi = Chinese journal of integrated traditional and Western medicine / Zhongguo Zhong xi yi jie he

International Journal of Pharmacy and Pharmaceutical Sciences<br>International Journal of Preventive Medicine<br>International Journal of Radiation Oncology, Biology, Physics<br>International Journal of Spine Surgery<br>International Journal of Stroke<br>International Journal of Tuberculosis and Lung Disease<br>International Journal of Urology<br>International Journal of Vascular Medicine<br>International Orthopaedics<br>INTERNATIONAL UROGYNECOLOGY JOURNAL<br>International Wound Journal<br>IOVS	xue hui, Zhongguo Zhong yi yan jiu yuan zhu ban<br>Zhongguo Zhong yao za zhi = Zhongguo zhongyao zazhi = China journal of Chinese materia medica<br>Zhonghua lao dong wei sheng zhi ye bing za zhi = Zhonghua laodong weisheng zhiyebing zazhi = Chinese journal of industrial hygiene and occupational diseases<br>Zhonghua liu xing bing xue za zhi = Zhonghua liuxingbingxue zazhi<br>Zhonghua wei chang wai ke za zhi = Chinese journal of gastrointestinal surgery<br>Zhonghua yu fang yi xue za zhi [Chinese journal of preventive medicine]<br>Zhonghua zhong liu za zhi [Chinese journal of oncology]

Table S4 Search terms to classify cost-utility and cost-benefit analyses

The following search terms were used to classify articles within our final database of full health economic evaluations according to study type. Searches were conducted in titles and abstracts. Search terms could classify an article as a cost-utility analysis, cost-benefit analysis, both, or neither. Articles in our database which did not contain search terms for cost-utility analyses or cost-benefit analyses were categorized as cost-effectiveness analyses. Question marks ("?") represent a single wildcard character or space.

Type of analysis	Search terms implemented in Excel database
**Cost-utility analysis**	Cost?utility<br>[Additionally, all results of DALY and QALY searches also included]
**CUA employing DALYs**	DALY, Disability?adjusted?life?year
**CUA employing QALYs**	QALY, Quality?adjusted?life?year, EQ?5D, SF?36, SF?12, SF?6D
**Cost-benefit analysis**	Cost?benefit, benefit?cost, net?benefit, net?monetary?benefit

Table S5 Search findings by database – all articles and databases

All searches were conducted on 3 May 2014, except for the LILACS database, which was searched on 12 May 2014. The first database listed identified the largest number of economic evaluations. Remaining databases are listed in order of those which identified the most additional economic evaluations beyond those already identified by other databases higher on the list.

**Database**	**Number of records identified by search**	**Number of economic evaluations meeting inclusion criteria**	**Sensitivity** (% of total economic evaluations)	**Specificity** (% of search results classified as economic evaluations)	**Additional economic evaluations** *(Beyond those found in databases higher on this list)*	**Cumulative %**
**Scopus**	9006	2409	85%	27%	2409	85%
**NHS EED**	3634	2280	80%	63%	314	96%
**Medline**	7566	2254	79%	30%	65	98%
**Global Health**	2219	691	24%	31%	19	99%
**Wiley HEED**	2175	1707	60%	78%	13	99%
**Web of Science**	8738	1852	65%	21%	6	99%
**Biosis**	2643	834	29%	32%	5	100%
**Embase**	7558	2217	78%	29%	4	100%
**Cinahl**	2580	1097	39%	43%	4	100%
**Scielo**	162	53	2%	33%	3	100%
**PsycInfo**	808	183	6%	23%	1	100%
**EconLit**	186	42	1%	23%	1	100%
**Lilacs**	132	42	1%	32%	0	100%
**TOTAL**	22807	2844				

Table S6 Search findings by database – excluding NHS EED and Wiley HEED

All searches were conducted on 3 May 2014, except for the LILACS database, which was searched on 12 May 2014. The first database listed identified the largest number of economic evaluations. Remaining databases are listed in order of those which identified the most additional economic evaluations beyond those already identified by other databases higher on the list. As NHS EED ceased to update records from March 2015 and Wiley HEED ceased to be available from the end of 2014, they have been placed at the bottom of the list to permit examination of available databases.

**Database**	**Number of records identified by search**	**Number of economic evaluations meeting inclusion criteria**	**Sensitivity** (% of total economic evaluations)	**Specificity** (% of search results classified as economic evaluations)	**Additional economic evaluations** *(Beyond those found in databases higher on this list)*	**Cumulative %**
**Scopus**	9006	2409	85%	27%	2409	85%
**Medline**	7566	2254	79%	30%	156	90%
**Global Health**	2219	691	24%	31%	35	91%
**Web of Science**	8738	1852	65%	21%	22	92%
**Embase**	7558	2217	78%	29%	14	93%
**Biosis**	2643	834	29%	32%	7	92%
**Cinahl**	2580	1097	39%	43%	5	93%
**Scielo**	162	53	2%	33%	3	93%
**EconLit**	186	42	1%	23%	1	93%
**PsycInfo**	808	183	6%	23%	1	93%
**Lilacs**	132	42	1%	32%	0	93%
(NHS EED)	*(3634)*	*(2280)*	*(80%)*	*(63%)*	*(183)*	*(100%)*
(Wiley HEED)	*(2175)*	*(1707)*	*(60%)*	*(78%)*	*(8)*	*(100%)*
**TOTAL**	22807	2844				

Table S7 Search findings by database – only articles studying low- and middle-income countries, excluding NHS EED and Wiley HEED

All searches were conducted on 3 May 2014, except for the LILACS database, which was searched on 12 May 2014. The first database listed identified the largest number of economic evaluations. Remaining databases are listed in order of those which identified the most additional economic evaluations beyond those already identified by other databases higher on the list. L&MIC: Low- and middle-income country. As NHS EED ceased to update records from March 2015 and Wiley HEED ceased to be available from the end of 2014, they have been placed at the bottom of the list to permit examination of available databases.

**Database**	**Number of records identified by search**	**Number of L&MIC economic evaluations meeting inclusion criteria**	**Sensitivity** (% of total L&MIC economic evaluations)	**Specificity** (% of search results classified as L&MIC economic evaluations)	**Additional L&MIC economic evaluations** *(Beyond those found in databases higher on this list)*	**Cumulative %**
**Scopus**	9006	428	81%	5%	428	81%
**Medline**	7566	380	72%	5%	40	89%
**Global Health**	2219	287	54%	13%	23	93%
**Biosis**	2643	181	34%	7%	4	94%
**Embase**	7558	403	76%	5%	4	95%
**Web of Science**	8738	316	60%	4%	3	95%
**Cinahl**	2580	119	23%	5%	1	95%
**Scielo**	162	43	8%	27%	1	96%
**Lilacs**	132	39	7%	30%	0	96%
**PsycInfo**	808	21	4%	3%	0	96%
**EconLit**	186	6	1%	3%	0	96%
(NHS EED)	*(3634)*	*(378)*	*(72%)*	*(10%)*	*(21)*	*(100%)*
(Wiley HEED)	*(2175)*	*(294)*	*(56%)*	*(14%)*	*(2)*	*(100%)*
**TOTAL**	47407	527				

Table S8 Journal concentration by income group of countries studied

	LICs	Lower-MICs	Upper-MICs	HICs	All
**Total articles**	104	121	391	2350	2844
**Avg articles per journal**	2.4	2.0	1.7	2.9	2.9
**Total journals**	44	61	226	802	967
**% articles in top 10 journals**	62%	52%	27%	22%	21%
**Total articles in top 10 journals**	64	63	105	509	600
**% articles in top 20 journals**	77%	66%	38%	29%	29%
**Total articles in top 20 journals**	80	80	147	684	813

Table S9 Number and proportion of economic evaluations by type and income group

In this table, "cost-effectiveness analyses" refers to articles meeting our definition of a full economic evaluation but not containing any keywords to define it more specifically as a cost-utility or cost-benefit analysis. Articles can be classified as both cost-utility and cost-benefit analyses if they contain keywords for both. DALY: disability-adjusted life year, QALY: quality-adjusted life-year.

	**Income group studied**											
	LICs		Lower-MICs		Upper-MICs		HICs		Multiple income groups		Total	
**Type of analysis**	N	%	N	%	N	%	N	%	N	%	N	%
**Cost-utility analysis (only)**	51	49%	63	52%	172	44%	1391	59%	39	61.9%	**1605**	56%
DALY	44	42%	40	33%	49	13%	34	1%	28	44.4%	**112**	4%
QALY	7	7%	22	18%	120	31%	1332	57%	10	15.9%	**1465**	52%
**Cost-benefit analysis (only)**	5	5%	3	2%	13	3%	60	3%	1	1.6%	**79**	3%
**Cost-benefit & cost-utility analysis**	1	1.0%	3	2.5%	10	2.6%	57	2.4%	1	1.6%	**68**	2%
**Cost-effectiveness analysis**	47	45%	52	43%	196	50%	842	36%	22	34.9%	**1092**	38%
**Total**	**104**	100%	**121**	100%	**391**	100%	**2350**	100%	**63**	100.0%	**2844**	100%
**%**	3.7%		4.3%		13.7%		82.6%		2.2%		100.0%	

References

LIBERATI, A., ALTMAN, D. G., TETZLAFF, J., MULROW, C., GOTZSCHE, P. C., IOANNIDIS, J. P., CLARKE, M., DEVEREAUX, P. J., KLEIJNEN, J. & MOHER, D. 2009. The PRISMA statement for reporting systematic reviews and meta-analyses of studies that evaluate health care interventions: explanation and elaboration. *J Clin Epidemiol,* 62**,** e1-34.

WORLD HEALTH ORGANIZATION. 2011. *International statistical classification of diseases and related health problems, 10th revision, edition 2010,* Geneva, World Health Organization.

WORLD HEALTH ORGANIZATION. 2014. Global Health Estimates 2014 Summary Tables: DALY by cause, age and sex, by World Bank income group category, 2000-2012. Geneva: World Health Organization.
